# A neutralizing nanobody targeting a conserved lateral patch on HA1 confers protection against multiple H7 avian influenza viruses

**DOI:** 10.1128/jvi.00563-26

**Published:** 2026-06-11

**Authors:** Siqi Xu, Qinying Zhang, Xueer Xie, Mengruo Zhou, Yunxia Chen, Yutong Liu, Chenying Luo, Qi Zhang, Han Zheng, Saixiang Feng, Ming Liao

**Affiliations:** 1 College of Veterinary Medicine, South China Agricultural University 12526, Guangzhou, China; 2 College of Life Sciences, South China Agricultural University 12526, Guangzhou, China; 3 Institute of Animal Health, Guangdong Academy of Agricultural Sciences 117866, Guangzhou, China; 4 Zhongkai University of Agricultural and Engineering, Guangzhou, China; Emory University School of Medicine, Atlanta, Georgia, USA

**Keywords:** therapeutic, neutralizing nanobody, HA1 protein, yeast two-hybrid, avian influenza virus

## Abstract

Human infections with H7 avian influenza viruses (AIVs) have been documented globally, involving multiple subtypes and geographic regions. However, effective therapeutics targeting H7 influenza viruses remain limited. Here, a panel of nanobodies targeting the HA1 domain of hemagglutinin (HA) was identified by yeast two-hybrid (Y2H) screening, and six candidates were subsequently validated to exhibit hemagglutination inhibition (HI) activity. Of these, a subset also displayed virus microneutralization (MN) activity, while all showed binding activity in ELISA assays. Among them, Nb74 exhibited inhibitory activity against four Chinese recombinant vaccine-matched strains (Rv1–Rv4), which were generated based on the HA sequences of the corresponding inactivated vaccine strains H7-Re1 to H7-Re4. The HI-IC_50_ values were 0.23, 0.57, 3.65, and 43.75 µg/mL, respectively, and the MN-IC_50_ values for Rv1–Rv3 were 0.02, 0.06, and 1.09 µg/mL. It also retained activity against diverse clinical isolates although HI potency varied among strains. In mouse challenge experiments, intratracheal administration of Nb74 conferred robust protection, achieving 100% and 80% survival against Rv1 and Rv2, respectively, when administered prophylactically (2 mg/kg) or therapeutically (4 mg/kg). Treated mice showed accelerated body weight recovery, reduced lung viral load, and alleviated pulmonary pathology. Mechanistic analyses indicated that Nb74 neutralizes virus by blocking viral attachment to the host. Furthermore, combined hydrogen-deuterium exchange mass spectrometry (HDX-MS) with escape mutant analysis mapped its epitope to a conserved lateral patch on the HA1 subunit, consistent with a conformational epitope. Overall, these results demonstrate the therapeutic promise of intratracheally delivered Nb74 and provide insights for H7 AIVs vaccine design.

**IMPORTANCE:**

H7 avian influenza viruses (AIVs), including both low- and highly pathogenic strains, pose a persistent threat to poultry production and public health, while continuous antigenic evolution of the HA1 subunit undermines vaccine effectiveness. Broadly neutralizing agents against H7 AIVs are urgently needed for pandemic preparedness, yet nanobody-based therapeutics remain largely unexplored. In this study, we identified Nb74, a nanobody that inhibits hemagglutination and neutralizes multiple H7 strains. Importantly, intratracheal delivery of Nb74 conferred protective efficacy, supporting respiratory administration of nanobody therapeutics. Mechanistic analyses show that Nb74 blocks viral attachment by recognizing a conserved conformational epitope within a lateral patch of the HA1 subunit. These findings reveal a conserved and functionally vulnerable region on H7 hemagglutinin (HA) and support the development of nanobody-based antivirals and improved vaccine strategies against emerging H7 AIV threats.

## INTRODUCTION

Avian influenza viruses (AIVs) are classified as low- or highly pathogenic (LPAIVs and HPAIVs), with HPAIVs predominantly arising from the H5 and H7 subtypes. In poultry, H7 LPAIVs can spread sub-clinically and subsequently acquire mutations that convert them into HPAIVs, causing severe systemic disease and elevated mortality (1). H7 AIVs are widely distributed globally and have caused sporadic human infections across multiple regions. Transmission to humans typically occurs through exposure to infected poultry, and most cases present with ocular disease or mild respiratory symptoms (1, 2). However, severe disease and fatal outcomes have also been reported for H7N2, H7N3, and H7N7 in regions such as Europe and North America, highlighting the zoonotic potential of H7 viruses (3–5). Phylogeographic studies further indicate that H7 viruses exhibit global dissemination patterns with distinct Eurasian and North American lineages, emphasizing the need for coordinated global surveillance within a One Health framework (6). Between 1959 and 2019, 1687 individuals worldwide were infected with H7 strains, resulting in 617 deaths. Among the recorded cases, 1,568 infections and 616 fatalities were attributed to H7N9 in China since its emergence in 2013, whereas only a single human infection with H7N4 has been reported (7, 8). Although no human cases of H7N9 infection have been recorded since March 2019, the virus remains detectable in the environment and poultry (9–11). Continuous antigenic drift and shift promote the emergence of highly transmissible variants and compromise vaccine-induced immunity (12). Vaccination remains the primary preventive strategy; however, vaccine production typically requires at least 6 months, limiting responsiveness to emerging outbreaks. In addition, vaccine efficacy is often reduced in immunocompromised and elderly populations, further constraining its protective impact. Moreover, the resistance to antiviral drugs, including neuraminidase inhibitors such as oseltamivir and zanamivir, further underscores the need for alternative antiviral strategies.

Passive immunotherapy represents an alternative strategy that can provide rapid protection. Antibody-based therapeutics have demonstrated remarkable efficacy against several viral infections, including respiratory syncytial virus and Zika virus (13–15). For influenza, the major surface glycoprotein hemagglutinin (HA) serves as the principal target for neutralizing antibodies, as it mediates viral entry and immune recognition. Within HA, the HA1 subunit forms a globular head containing critical antigenic sites, which is, therefore, the primary focus of neutralizing antibodies (16). Although numerous neutralizing antibodies targeting H7 HA have been widely characterized, ongoing antigenic evolution of H7 viruses undermines vaccine effectiveness and may reduce the durability of antibody-based interventions (16–24). Moreover, while many of these antibodies demonstrate potent antiviral activity in preclinical studies, their practical application is often limited by the need for intravenous or intraperitoneal administration (21, 23, 25, 26). However, the repeated updates of vaccine strains in China, from Re1 to Re4 between 2013 and 2024, highlight the rapid evolution of H7 viruses (10). Therefore, neutralizing antibodies that are active against diverse emerging H7 influenza strains and amenable to alternative delivery routes remain an important goal.

Beyond conventional antibodies, nanobodies have recently emerged as versatile tools for diagnostics and therapeutics. These ~15 kDa fragments are derived from the variable domain of camelid heavy-chain antibodies (27). Nanobodies are characterized by small molecular size, high stability, low immunogenicity, and the unique ability to recognize conserved or cryptic epitopes (13, 28, 29). Moreover, their efficient tissue penetration and low-cost production further enable practical translational implementation. Although nanobody-based therapeutics have shown promise against severe acute respiratory syndrome coronavirus 2 (SARS-CoV-2) and other pathogens (30–33), their application against epidemic H7 viruses remains largely unexplored, emphasizing the need to evaluate nanobody-based strategies for zoonotic influenza (25, 28, 34–37).

Notably, previous yeast display studies have systematically mapped the epitopes of H7-targeting nanobodies, revealing both cross-reactive and strain-specific antigenic sites (38), and Huang et al. employed a yeast two-hybrid (Y2H) screening strategy to identify H7-specific nanobodies (39). However, these studies primarily focused on nanobody identification and *in vitro* characterization, with limited investigation into the neutralization mechanisms or *in vivo* efficacy of H7-targeting nanobodies. Thus, whether and how these nanobodies achieve functional neutralization *in vivo* remains largely unexplored.

To address this gap, we have identified nanobody candidates against the HA1 domain of H7-Rv1 using yeast two-hybrid (Y2H), and six candidates were validated for hemagglutination inhibition (HI) activity. Among them, Nb74 displayed potent neutralizing activity against multiple H7 strains and provided significant protection in a mouse challenge model following intratracheal administration. Mechanistic studies demonstrated that Nb74 blocks viral attachment, and further epitope mapping by hydrogen-deuterium exchange mass spectrometry (HDX-MS) and escape mutant analyses revealed that it targets a conserved lateral patch on the HA1 subunit. This study identifies a promising therapeutic approach and yields insights that may guide the development of broadly protective influenza vaccines.

## RESULTS

### Selection of H7-Rv1 nanobodies

**Fig 1:**
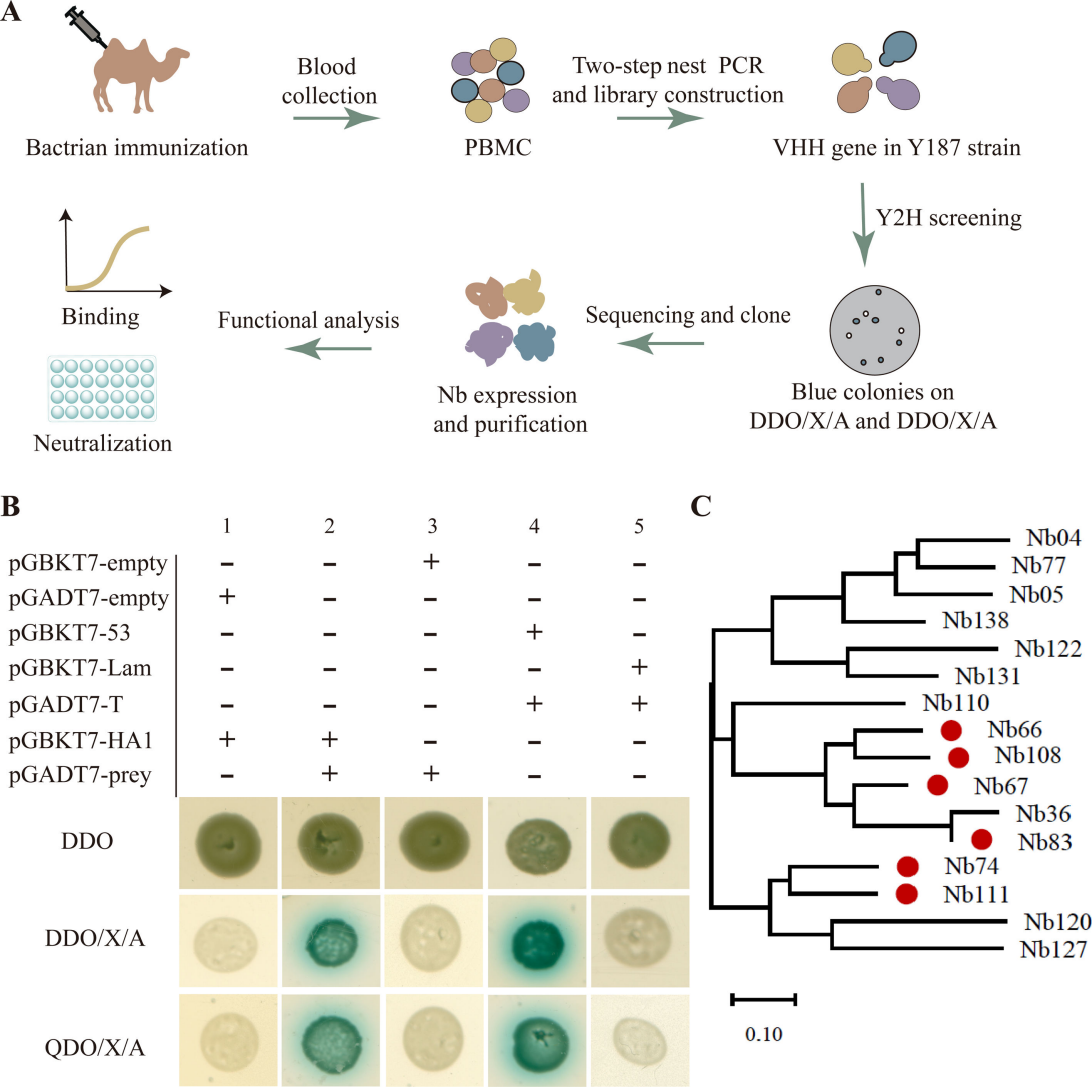
Nanobody generation and selection. (**A**) Overview of the yeast two-hybrid (Y2H) screening process. Nanobodies specific to the Rv1-HA1 protein were isolated from blue colonies and further validated for binding and neutralization activities. (**B**) Isolation of positive nanobodies from the Y2H mating assay. Presence (+) and absence (−) of plasmids are indicated. White colonies on DDO and no growth on DDO/X/A and QDO/X/A plates indicated no interaction, while the formation of blue colonies on DDO/X/A and QDO/X/A, along with white colonies on DDO, indicated a positive interaction between bait and prey protein. DDO represented double dropout medium lacking tryptophan and leucine. DDO/X/A represented DDO plates supplemented with X-α-Gal and aureobasidin A. QDO/X/A represented quadruple dropout medium lacking tryptophan, leucine, histidine, and adenine supplemented with X-α-Gal and aureobasidin A. (**C**) Phylogenetic analysis of HA1-specific nanobody sequences identified by yeast two-hybrid screening. Phylogenetic tree of nanobody sequences obtained from yeast two-hybrid screening based on blue colony selection targeting the HA1 protein. The phylogenetic tree was built using the maximal likelihood method, with the scale bar indicated a 10% difference in amino acid identity.

To generate H7-specific nanobodies, a camel was immunized with H7-Rv1 vaccine, and peripheral blood mononuclear cells (PBMCs) were collected for library construction. The extracted RNA was used to amplify the variable domains of heavy-chain antibodies (VHH), which were subsequently cloned into pGADT7-Rec to generate pGADT7-prey constructs. These constructs were then transformed into the yeast strain Y187 to establish a yeast two-hybrid (Y2H) library (Fig. 1A). The final library titer was approximately 3.05 × 10^7^ CFU/mL, with a size of ~5.82 × 10^8^ CFU (Fig. S1A). Sequence analysis confirmed the correct insertion of VHH fragments, with 24 of 25 colonies displaying unique sequences, of which 23 (92%) were in frame with the activation domain (Fig. S1B and C). These results indicate that the library is of high quality and possesses sufficient diversity for subsequent screening

The bait yeast strain Y2HGold containing pGBKT7-HA1 exhibited a white phenotype on SD/–Trp/X-alpha-Gal (synthetic defined medium lacking tryptophan supplemented with X-α-Gal) but failed to form colonies on SD/–Trp/X-alpha-Gal/AbA (synthetic defined medium lacking tryptophan supplemented with X-α-Gal and aureobasidin A) agar plates, excluding the self-activation of the HA1 protein in yeast (Fig. S1D). Colony number and size were comparable between pGBKT7-HA1 and pGBKT7-empty on SD/–Trp (synthetic defined medium lacking tryptophan), indicating the absence of toxicity (Fig. S1D). In addition, co-transformation with pGBKT7-HA1 and pGADT7-empty produced no positive signal (Fig. 1B, lane 1), confirming the absence of nonspecific interactions. These results demonstrate that Re1-HA1 is nontoxic and non-self-activating and, therefore, suitable for library screening.

Following 20 h of mating between the bait strain and the Y2H library, potential positive colonies were selected stepwise on DDO/X/A (double dropout medium lacking tryptophan and leucine supplemented with X-α-Gal and aureobasidin A) and further screened on QDO/X/A (quadruple dropout medium lacking tryptophan, leucine, histidine, and adenine supplemented with X-α-Gal and aureobasidin A) plates. Twenty blue colonies from QDO/X/A plates were sequenced, leading to the identification of 16 nanobodies (Table S1 and Fig. S1E). Then, the specificity of the interaction between nanobodies and HA1 was confirmed by co-transformation of yeast cells with pGBKT7-HA1 and pGADT7-prey constructs. Representative positive clones harboring pGBKT7-HA1 and pGADT7-prey are shown in Fig. 1B (lane 2). In contrast, no signal was detected when positive prey was co-expressed with pGBKT7-empty in Y2H (lane 3), further supporting the specific interaction between Re1-HA1 and the isolated VHHs. Appropriate positive and negative controls were included in lanes 4 and 5, respectively. Phylogenetic analysis of the amino acid sequences from blue colonies was performed using MEGA11 with the maximum likelihood method (Fig. 1C).

### Nanobody expression and characterization

**Fig 2:**
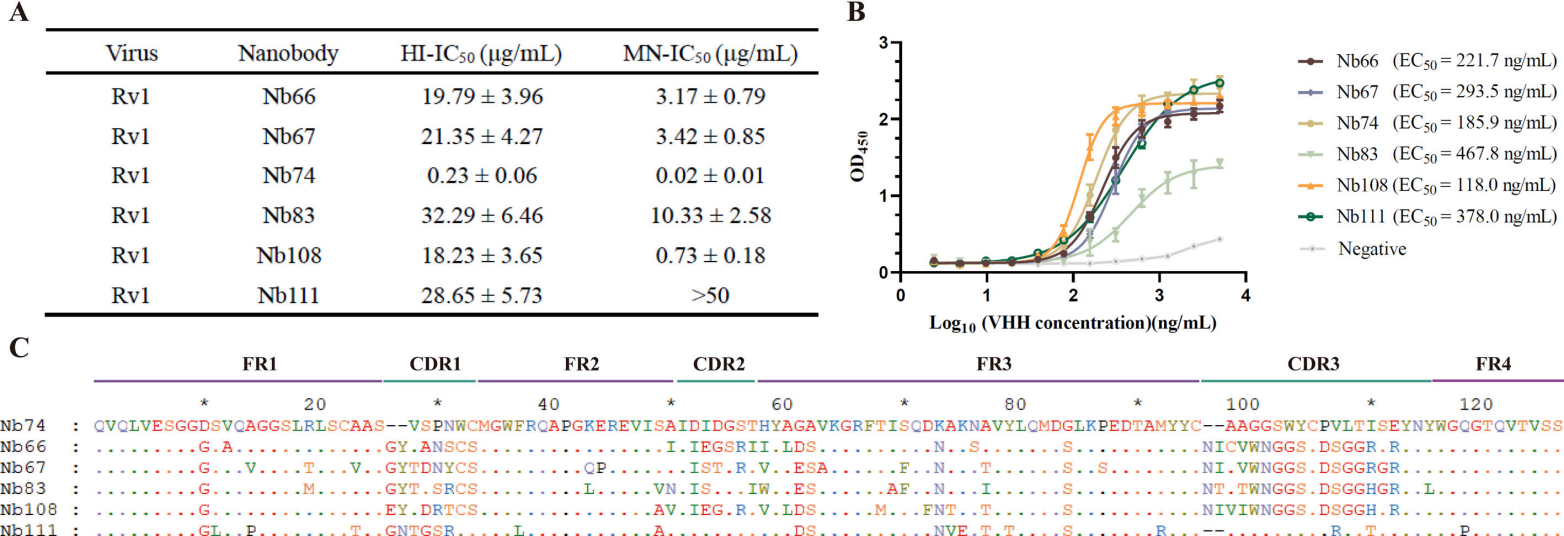
Characterization of neutralization and binding activity, and sequence alignment of HA1-specific nanobodies. (**A**) Neutralization assay results for nanobodies against Rv1. The results represented the mean ± SEM of three independent experiments. HI, hemagglutination inhibition; MN, microneutralization. Concentrations more than 50 µg/mL were marked as negative. (**B**) Enzyme-linked immunosorbent assay of nanobody binding to recombinant Rv1-HA1 protein. The negative nanobody did not specifically bind to Rv1-HA1. Error bars represented the mean ± SEM from triplicate analyses. (**C**) Amino acid sequence alignment of nanobodies exhibiting HI activity. Complementarity-determining regions (CDRs) and framework regions (FRs) were marked above the panel. Dashed lines represented missing residues; dots indicated residues identical to those in Nb74.

To evaluate the functional activity of nanobodies identified from the Y2H screening, their binding and antiviral properties were assessed by hemagglutination inhibition (HI), microneutralization (MN), and ELISA assays. Sixteen nanobodies were expressed in *Pichia pastoris* X33 with either a 6 × His or Twin-Strep tag and purified to homogeneity, as confirmed by 15% SDS-PAGE. Among them, six nanobodies exhibiting HI activity, including Nb66, Nb67, Nb74, Nb83, Nb108, and Nb111, and are shown in the gel, highlighting their purity and expected molecular weight (Fig. S1F). Among them, Nb74 exhibited the strongest neutralizing activity, with the lowest average HI-IC_50_ (0.23 ± 0.06 µg/mL) and MN-IC_50_ (0.02 ± 0.01 µg/mL) values (Fig. 2A). In contrast, Nb111 showed no detectable MN activity even at concentrations up to 50 µg/mL, likely due to its relatively weak hemagglutination inhibition potency, although the specific stage affected remains unclear. The average HI-IC_50_ and MN-IC_50_ values for the other nanobodies are shown in Fig. 2A. The HA1 protein, derived from the HA sequences of the corresponding vaccine strains, was expressed in an insect cell-based expression system and subsequently evaluated for binding activity (Fig. S1G and Table S2). Binding analysis by ELISA revealed that Nb108 had the highest affinity for Rv1-HA1 (EC_50_ = 118.0 ng/mL), followed by Nb74 (EC_50_ = 185.9 ng/mL) (Fig. 2B). In contrast, Nb83 and Nb111 showed poor reactivity in HI, MN, and ELISA assays (Fig. 2A and 2B). The framework regions (FRs) and complementarity-determining regions (CDRs) of the six nanobodies are illustrated in Fig. 2C, revealing substantial variability within the CDR regions.

### Neutralizing potency and breadth of nanobodies

**Fig 3:**
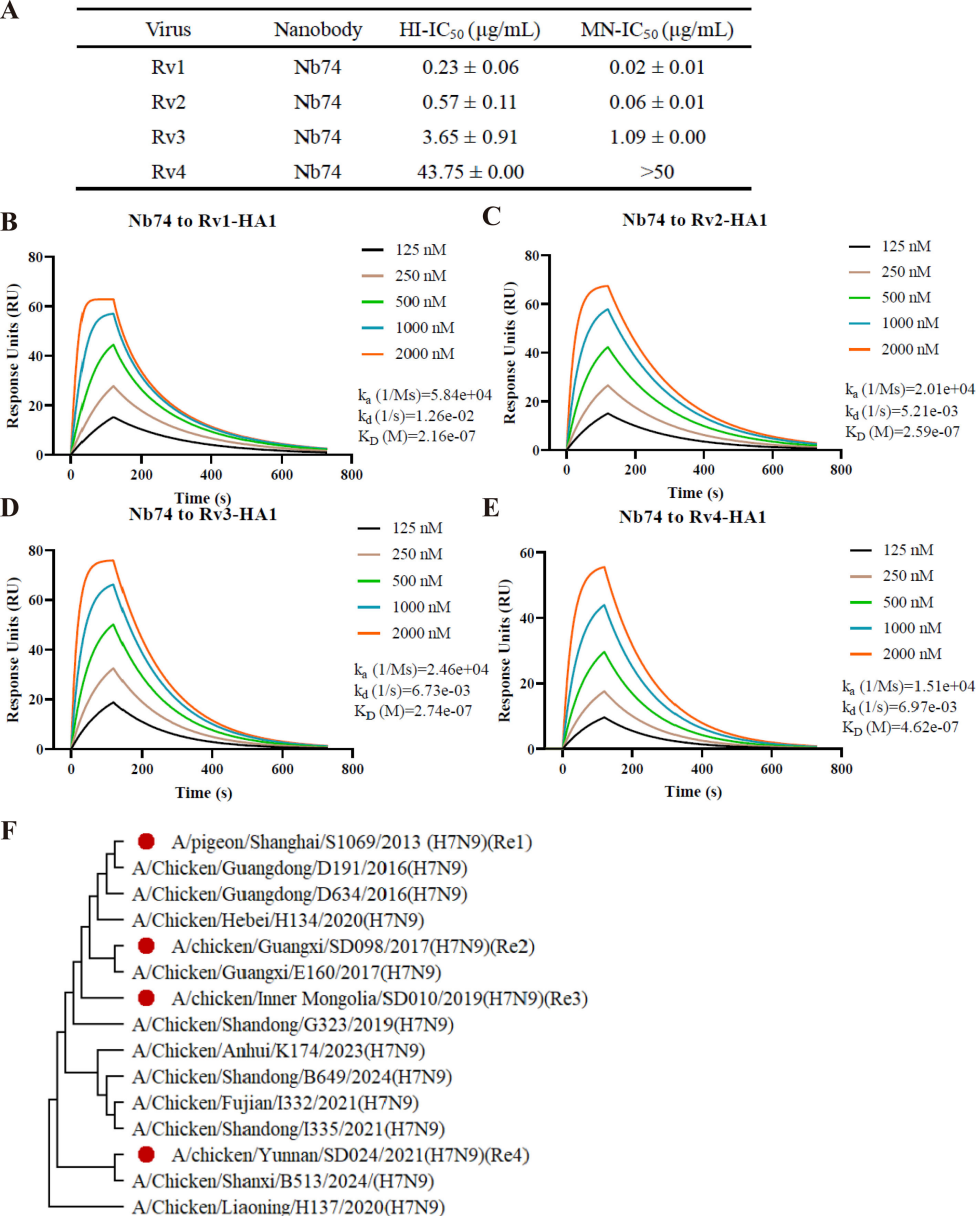
Reactivity profile of Nb74 against H7 viruses. (**A**) Neutralization activity against representative H7 viruses generated from vaccine strain HA recombinants. Nb74 exhibited neutralizing activity against most tested viruses, except for Rv4 in the MN assay. Values exceeding 50 µg/mL were considered negative. Data are presented as mean ± SEM of three independent experiments. (**B–E**) SPR sensorgrams depicting the biomolecular binding of Nb74 and HA1s. Nanobody binding activity was assessed over a concentration range from 2,000 nM to 125 nM, as indicated in each panel. Kinetic parameters are annotated in the corresponding panels. (**F**) Phylogenetic tree of the HA1 amino acid sequences from all viruses included in this study. The tree illustrates the genetic relationships among the tested strains used for evaluating Nb74 activity.

To further assess the neutralizing potency and breadth of the identified nanobodies, their activity was evaluated against multiple recombinant H7 strains. All nanobodies, with the exception of Nb74, displayed neutralizing activity restricted to Rv1, indicating limited breadth (Table S3). Nb74 demonstrated the broadest spectrum, inhibiting Rv1, Rv2, Rv3, and Rv4, with average HI-IC_50_ values of 0.23 ± 0.06, 0.57 ± 0.11, 3.65 ± 0.91, and 43.75 ± 0.00 µg/mL, respectively (Fig. 3A). Consistently, Nb74 neutralized Rv1, Rv2, and Rv3, with MN-IC_50_ values ranging from 0.02 to 1.09 µg/mL, but showed no activity against Rv4 at 50 µg/mL. Surface plasmon resonance (SPR) analysis confirmed strong binding of Nb74 to HA1 proteins from Rv1–Rv4 with *K*_D_ values of 2.16 × 10E−7 to 4.62 × 10E−7 M, corresponding to the highest affinity for Rv1 and the lowest for Rv4 (Fig. 3B through 3E and Table 1, raw SPR sensorgrams in Fig. S2A through S2D).

**TABLE 1:** Steady-state binding equilibrium analysis of Nb74 to different subtypes of H7-HA1^*a*^

Antigen	Analyte	*k*_a_ (1/Ms)	*k*_d_ (1/s)	*K*_D_ (M)
Re1-HA1	Nb74	5.84E + 04	1.26E−02	2.16E−07
Re2-HA1	Nb74	2.01E + 04	5.21E−03	2.59E−07
Rv3-HA1	Nb74	2.46E + 04	6.73E−03	2.74E−07
Rv4-HA1	Nb74	1.51E + 04	6.97E−03	4.62E−07

^*a*^ Characterization of Nb74 binding kinetics to various H7-HA1 protein subtypes. The data involved *K*_D_ (equilibrium dissociation constant), *k*_a_ (association rate constant), and *k*_d_ (dissociation rate constant), with *K*_D_ computed as *k*_d_/*k*_a_.

Nb74 retained strong neutralizing activity against H7 clinical isolates from 2013 to 2019, but its HI potency decreased against viruses emerging after 2020, as several post-2020 strains exhibited HI values approaching or exceeding 50 µg/mL (Table 2). Consistent with HI results, phylogenetic analysis indicated that HA1 sequence divergence among vaccine and clinical isolates reflects the variation in Nb74 inhibitory activity (Fig. 3F). Together, these results suggest that Nb74 retains substantial neutralizing activity against diverse H7 strains, with reduced efficacy against some viruses emerging after 2020.

**TABLE 2:** Hemagglutination inhibition (HI) results of Nb74 against clinical H7 isolates collected from the past decade^*a*^

Strain	HI-IC_50_ (µg/mL)
A/pigeon/Shanghai/S1069/2013 (H7N9) (Rv1)	0.23 ± 0.06
A/Chicken/Guangdong/D191/2016 (H7N9)	0.23 ± 0.06
A/Chicken/Guangdong/D634/2016 (H7N9)	0.28 ± 0.06
A/chicken/Guangxi/SD098/2017 (H7N9) (Rv2)	0.57 ± 0.11
A/Chicken/Guangxi/E160/2017 (H7N9)	0.91 ± 0.23
A/chicken/Inner Mongolia/SD010/2019 (H7N9) (Rv3)	3.65 ± 0.91
A/Chicken/Shandong/G323/2019 (H7N9)	4.56 ± 0.91
A/Chicken/Hebei/H134/2020 (H7N9)	0.91 ± 0.23
A/Chicken/Liaoning/H137/2020 (H7N9)	>50
A/chicken/Yunnan/SD024/2021 (H7N9) (Rv4)	43.75 ± 0.00
A/Chicken/Fujian/I332/2021 (H7N9)	>50
A/Chicken/Shandong/I335/2021 (H7N9)	43.75 ± 0.00
A/Chicken/Anhui/K174/2023 (H7N9)	18.23 ± 3.65
A/Chicken/Shandong/B649/2024 (H7N9)	>50
A/Chicken/Shanxi/B513/2024/ (H7N9)	>50

^*a*^ The table lists representative H7 strains and the corresponding HI-IC_50_ values of Nb74. HI-IC_50_ is defined as the nanobody concentration required for 50% inhibition of hemagglutination. Data were presented as mean ± SEM (*n* = 3).

### Prophylactic and therapeutic potential of Nb74 in mice

**Fig 4:**
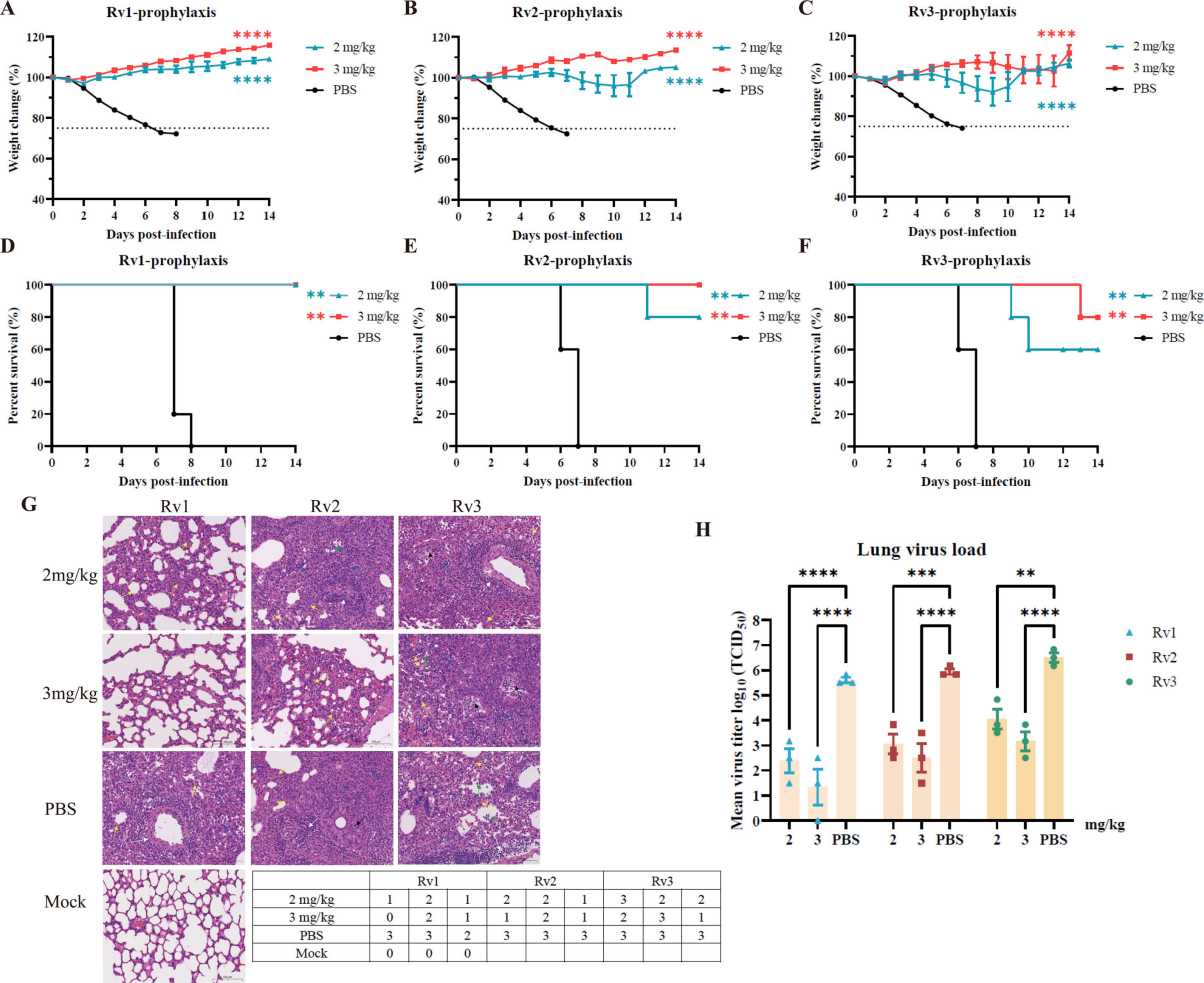
Prophylactic efficacy *in vivo*. (**A–F**) Weight loss (top panels, one-way analysis of variance) and survival (middle panels, Kaplan-Meier method) were recorded from day 0 to 14 and expressed as percentages (*n* = 5). (**G**) Lung histology. Lungs (*n* = 3) were examined by hematoxylin-eosin (HE) staining. Inflammatory cell infiltration (blue arrows) predominantly consisted of lymphocytes and macrophages; widened alveolar septa with infiltrated inflammatory cells are indicated by yellow arrows; compensatory alveolar dilation and infiltrated inflammatory cells in alveolar cavities are marked with green arrows; inflammatory cells surrounding blood vessels are indicated by white arrows; desquamated cell components in bronchial cavities are marked with black arrows. Lesion severity is shown at the bottom of panel G. The mock group did not receive the viral challenge. (**H**) Pulmonary viral load. Right lung tissues (*n* = 3) were homogenized for viral titers. Statistical analysis for comparison of virus load was performed using a 2-way ANOVA. Error bars indicated the mean ± SEM. Significant differences (*P* < 0.05) between groups and the PBS control are as follows: **, *P* < 0.01; ***, *P* < 0.001; and ****, *P* < 0.0001, as determined by analysis of variance using GraphPad Prism software.

To evaluate the *in vivo* protective efficacy of Nb74, its prophylactic and therapeutic potential was assessed in a mouse challenge model. In the prophylactic experiments, 6-week-old female SPF BALB/c mice (*n* = 8) received intratracheal administration of Nb74 (2 or 3 mg/kg) 24 h prior to intranasal challenge with 10 median lethal doses (MLD_50_) of Rv1, Rv2, or Rv3. Of these, five mice were randomly assigned for survival and body weight monitoring over a 14-day observation period, while the remaining three mice were used for viral load quantification and histopathological analysis and were euthanized at day 5 post-infection. Intratracheal administration of Nb74 (2–3 mg/kg) conferred strong protection against H7 challenge. Mice challenged with Rv1 and treated with 2 or 3 mg/kg Nb74 exhibited complete survival and marked weight recovery over a 14-day observation period (Fig. 4A through D). For Rv2, treatment with 3 mg/kg resulted in 100% survival with faster recovery from weight loss, while 2 mg/kg conferred 80% protection (Fig. 4B through E). Against Rv3, Nb74 provided 80% and 60% protection at 3 and 2 mg/kg, respectively, accompanied by modest weight recovery (Fig. 4C through F). In contrast, all the PBS-treated mice did not survive beyond day 8.

Histopathological analysis revealed dose-dependent mitigation of lung injury, with reduced inflammatory infiltration, vascular congestion, and alveolar exudation, as shown in Fig. 4G. On day 5 after infection, the virus concentration in the lung tissue (*n* = 3) was quantified using determination of tissue culture infectious dose (TCID_50_) assays. Consistently, viral titers in lung tissue decreased with increasing Nb74 dose, and these decreased titers were correlated with survival (Fig. 4H).

**Fig 5:**
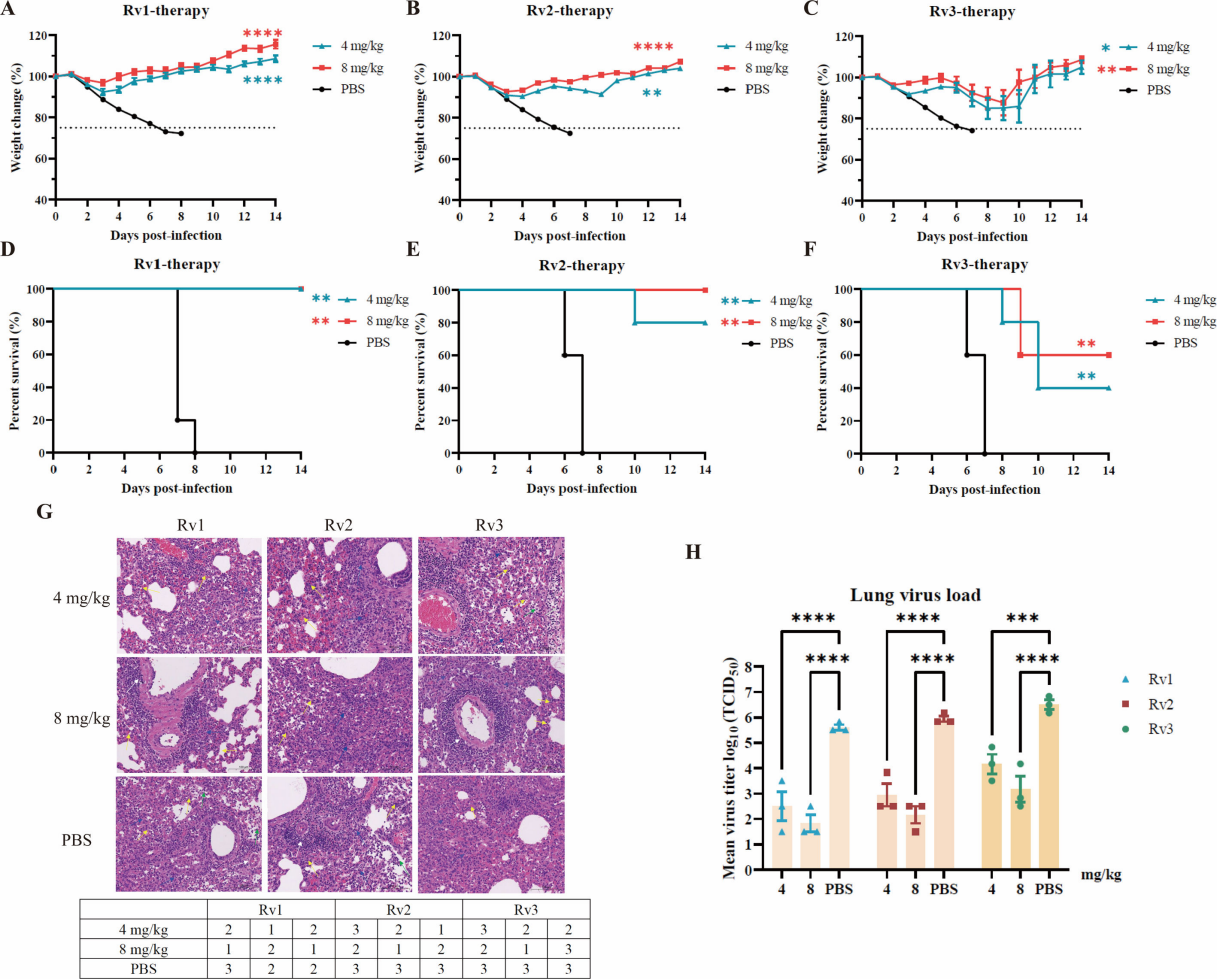
Therapeutic efficacy *in vivo*. (**A–F**) The top panels display weight loss, and the middle panels show survival curves, both tracked for 2 weeks. Weight change was analyzed using one-way ANOVA, while survival data were assessed using the Kaplan-Meier method. (**G**) Lung histology (H&E staining). Lungs (*n* = 3) from each group were used for pathological analysis. Inflammatory areas include swollen alveolar septa and infiltrated inflammatory cells (yellow arrows); invasion by inflammatory cells (mainly lymphocytes and macrophages) (blue arrows); compensatory alveolar dilation and inflammatory cell infiltration in alveolar cavities (green arrows); inflammatory cells surrounding blood vessels (white arrows); and desquamated cell components in bronchial cavities (black arrows). Lesion severity is detailed in the corresponding panel below. (**H**) Pulmonary viral load. Right lung tissue homogenization (*n* = 3) was used to determine the lung viral load. Data are presented as mean ± SEM. Statistical analysis using two-way ANOVA in GraphPad Prism software revealed significant differences (*P* < 0.05) from PBS controls, with ns indicating no significance, ** for *P* < 0.01, *** for *P* < 0.001, and **** for *P* < 0.0001, respectively.

For therapeutic evaluation, mice were intratracheally challenged with 10 MLD_50_ of virus and subsequently treated with Nb74 at 4 or 8 mg/kg 24 h post-infection, with the same group size and regimen as the prophylactic group. A single dose of Nb74 administered 24 h post infection provided full protection against Rv1 at 4 mg/kg, with greater weight recovery observed at 8 mg/kg (Fig. 5A through D). In the Rv2 challenge, 8 mg/kg achieved 100% survival, whereas 4 mg/kg conferred 80% protection with moderate weight recovery (Fig. 5B through E). In the Rv3 challenge, Nb74 provided partial protection (40%–60%) with more variable weight changes (Fig. 5C through F). In line with the prophylactic outcomes, Nb74 treatment significantly mitigated pulmonary pathology and decreased the lung viral burden in a dose-dependent manner, with higher doses corresponding to progressively milder histopathology and lower viral titers (Fig. 5G and H). Together, these results demonstrate that intratracheally delivered Nb74 provides potent prophylactic and therapeutic efficacy against H7 viruses, with the greatest efficacy observed against Rv1 and Rv2.

### Mechanisms of protection by Nb74

**Fig 6:**
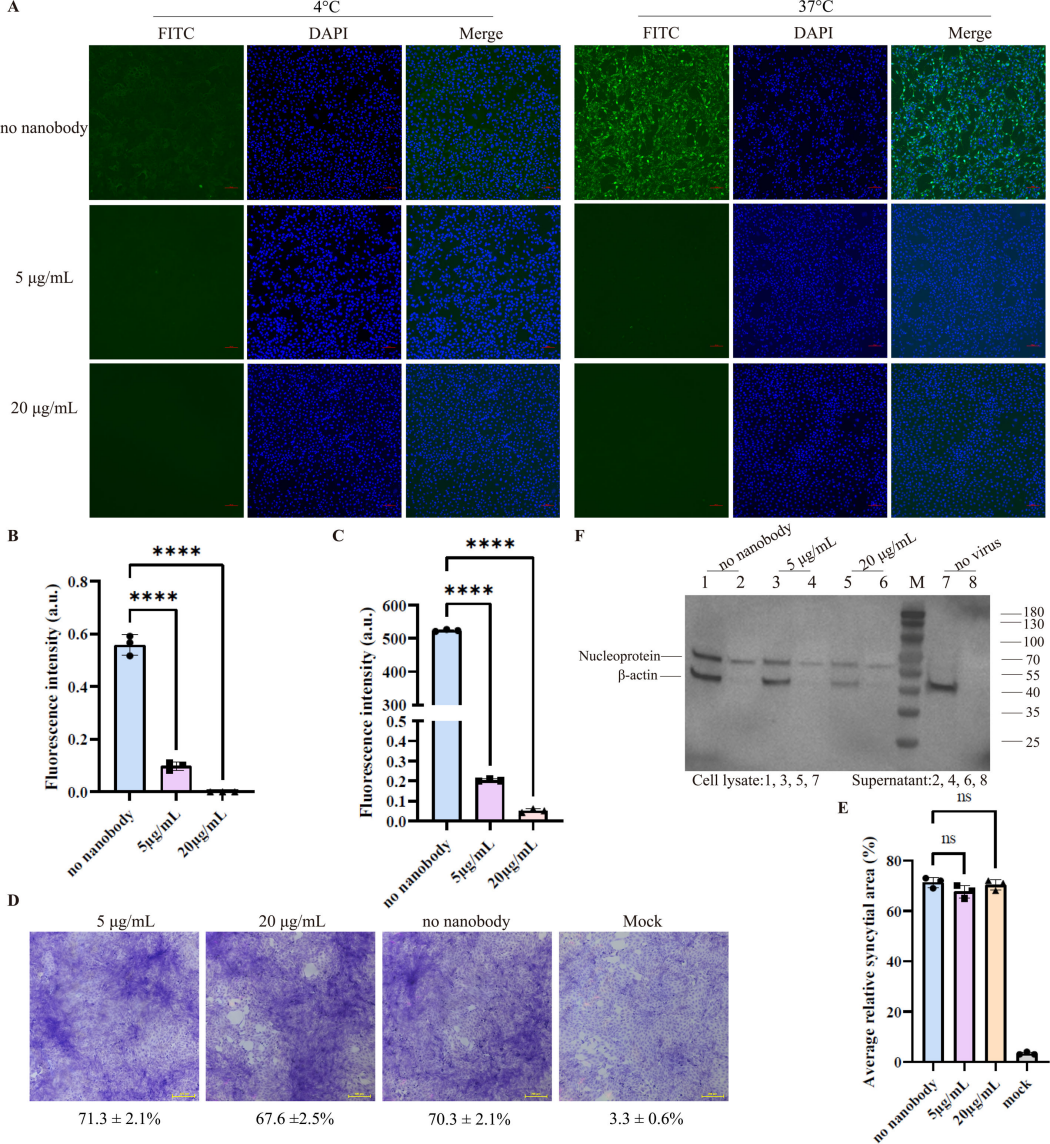
The mechanisms of antiviral activity. (**A**) Attachment inhibition of Nb74. Rv1 virus was preincubated with Nb74 and allowed to bind to MDCK cells at 4°C for 1 h. After washing, cells were either fixed immediately to assess viral attachment or further incubated at 37°C in nanobody-free medium for 16–18 h prior to analysis. Viral nucleoprotein (NP) was detected by immunofluorescence staining (green), and nuclei were counterstained with DAPI (blue). Merged images show the overlay of NP and nuclear signals. Nb74 was present during the binding step only. (**B and C**) Quantification of fluorescence intensity corresponding to NP staining (FITC channel) under the conditions shown in panel A. Fluorescence intensity measured after incubation at 4°C for attachment (**B**) or 37°C for infection (**C**). Fluorescence intensity (a.u.) was quantified using ImageJ and is presented as mean ± SD. Statistical analysis was performed using one-way ANOVA with appropriate multiple comparisons. **** for *P* < 0.0001. Results are representative of two independent experiments. (**D**) Effect of Nb74 on syncytia formation. MDCK cells infected with Rv1 were treated with Nb74 at the indicated concentrations prior to acid-induced membrane fusion. Cells were then fixed, Giemsa-stained, and analyzed by light microscopy. No significant difference in syncytium formation was observed between Nb74-treated and untreated groups, as indicated by comparable average relative syncytial areas, suggesting that Nb74 does not interfere with HA-mediated membrane fusion. No obvious syncytia were observed in the mock-treated group. The representative images are shown from three independent experiments. (**E**) Quantification of syncytia formation. Relative syncytial area (%) was measured to assess syncytia formation. Data are presented as mean ± SD (*n* = 3). Statistical analysis was performed using one-way ANOVA (ns, not significant). (**F**) Effect of Nb74 on viral egress. Following Rv1 infection, MDCK cells were incubated with Nb74 at the indicated concentrations. Viral NP levels (~57 kDa) in both cell lysates and supernatants were analyzed by Western blot. Comparable NP signals were detected in Nb74-treated and untreated samples, suggesting that Nb74 does not impact viral release. β-actin (~42 kDa) served as a loading control. Data are representative of two independent experiments.

Given the potent neutralizing activity and *in vivo* protective efficacy of Nb74, we next sought to elucidate the underlying mechanism of its antiviral action. Specifically, we investigated whether Nb74 inhibits viral attachment to host cells. Rv1 virus was preincubated with Nb74 and allowed to bind to MDCK cells at 4°C for 1 h, a condition that prevents viral internalization. After removal of unbound virus and nanobody, cells were either fixed immediately to assess attachment or further incubated at 37°C in nanobody-free medium for 16–18 h to evaluate subsequent infection. Immunofluorescence analysis showed that at the 4°C binding stage, nucleoprotein (NP) signals were markedly reduced at 5 µg/mL Nb74 and became barely detectable at 20 µg/mL compared to the no nanobody control, indicating that Nb74 effectively interferes with viral attachment to the cell surface (Fig. 6A, left panel). Consistent with this observation, NP signal at the 37°C infection stage was substantially decreased despite removal of Nb74 after the initial binding step (Fig. 6A, right panel), suggesting that inhibition at the attachment step is sufficient to impair downstream infection. To further substantiate these observations, fluorescence intensity was quantitatively analyzed using ImageJ. As shown in Fig. 6B and C, Nb74 treatment resulted in a significant reduction in fluorescence intensity at both 4°C and 37°C compared to the no-nanobody control (*P* < 0.0001). These quantitative results are consistent with the qualitative imaging data and further support that Nb74 efficiently blocks viral attachment and consequently suppresses subsequent infection.

To further delineate the mechanism of Nb74-mediated antiviral activity, its effects on membrane fusion were evaluated. MDCK cells infected with Rv1 and treated with Nb74 at 0, 5, or 20 µg/mL exhibited comparable levels of syncytium formation, as reflected by similar mean relative syncytial areas (71.3% ± 2.1%, 67.6% ± 2.5%, and 70.3% ± 2.1%, respectively) across groups (Fig. 6D), and quantified by measuring the relative syncytial area (Fig. 6E). No significant differences were observed among the no-nanobody, 5 µg/mL, and 20 µg/mL Nb74-treated groups. A minimal level of syncytia was occasionally observed in the mock-treated group (3.3% **±** 0.6%). These results indicate that Nb74 does not significantly interfere with HA-mediated membrane fusion.

To further assess whether Nb74 affects later stages of the viral life cycle, western blot (WB) analysis showed comparable levels of viral NP (~57 kDa) in both cell lysates and culture supernatants in the presence or absence of Nb74. These findings suggest that Nb74 does not affect viral production or release (Fig. 6C). Together, these data indicate that the antiviral activity of Nb74 is mediated predominantly through blockade of viral attachment rather than interference with fusion or egress.

### Convergent identification of the Nb74 epitope by Western blot, escape mutants, and hydrogen-deuterium exchange mass spectrometry

**Fig 7:**
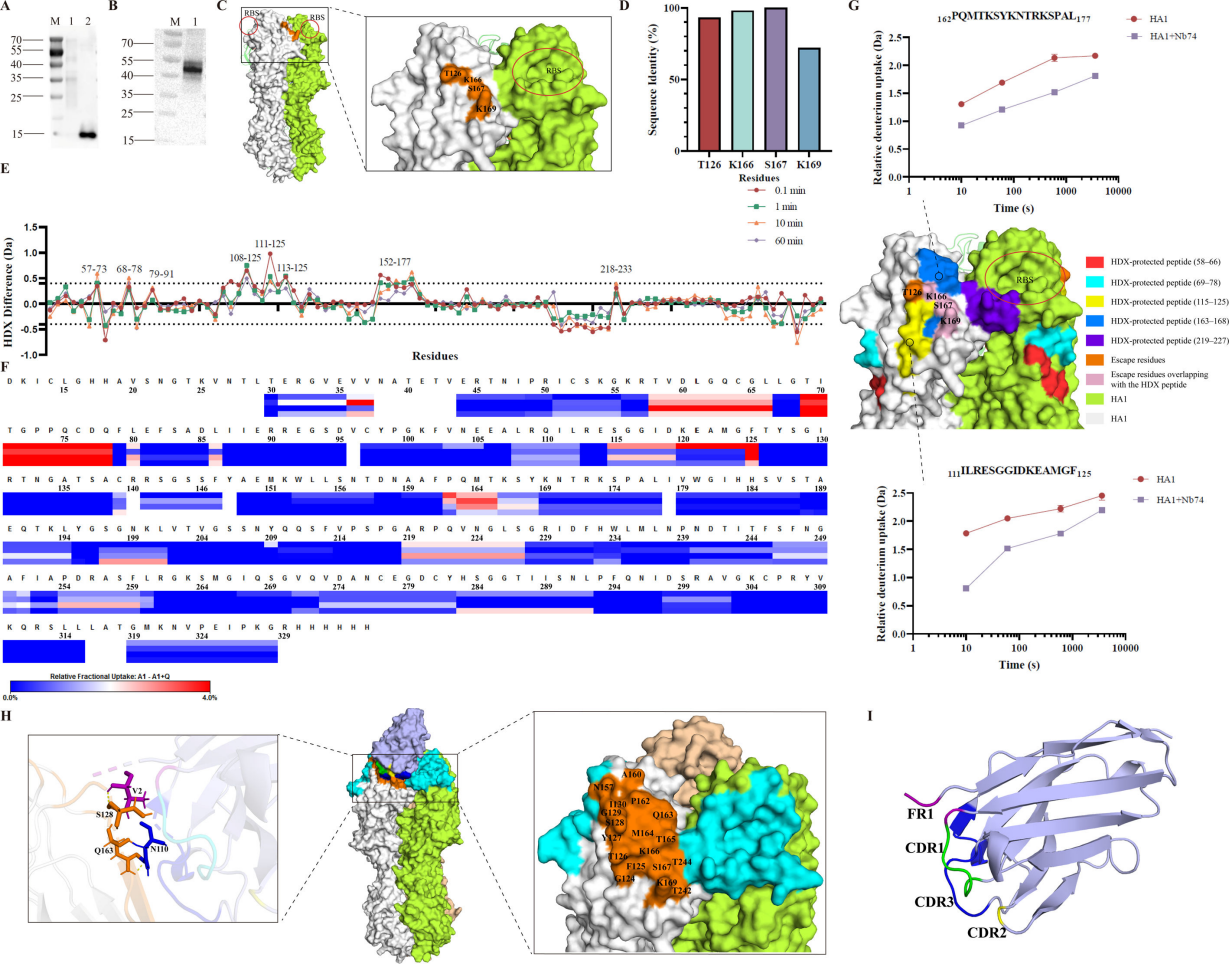
Convergent mapping of a conserved epitope on the lateral patch of the HA head recognized by Nb74. (**A**) Conformation-dependent epitope recognition. Nb74 failed to bind denatured Rv1-HA1 (lane 1), whereas Strep-tag detection confirms Nb74 (lane 2). (**B**) Validation of denatured Rv1-HA1. A single band was detected at approximately 40 kDa, consistent with the expected molecular weight. (**C**) Escape mutation mapping. Escape-associated residues are mapped onto the HA trimer (orange); protomers are shown in gray and green, and the receptor-binding site (RBS) are indicated by red circles. (**D**) Conservation analysis. Conservation of escape-associated residues across H7 HA amino sequences is shown as residue frequencies. (**E**) Hydrogen-deuterium exchange (HDX) profile. Differential deuterium uptake (ΔHDX) between free and Nb74-bound HA1 is shown across four labeling time points. Peptides are ordered from the N- to C-terminus. Regions exhibiting positive ΔHDX values correspond to decreased exchange, and negative values reflect increased exchange. Data represent the mean of two independent measurements. Dotted lines indicate the significance threshold for all peptides, defined as approximately 0.4 Da based on replicate analyses. (**F**) HDX heat map. Deuterium uptake differences across HA1 are shown, with red indicating decreased exchange and blue indicating increased exchange upon Nb74 binding. (**G**) Integrated epitope mapping of Nb74 on the HA trimer. Peptides exhibiting reduced deuterium uptake upon Nb74 binding are highlighted on the HA trimer structure. Regions that overlap with or are proximal to escape-associated residues are considered part of the epitope. Representative deuterium exchange kinetics for selected peptides are shown as mean ± SD. (**H**) Structural model of the Nb74-HA complex. The HA trimer is shown as a surface (gray, light green, and wheat), with Nb74 in light blue; the receptor-binding site (RBS) are highlighted in cyan. Left: cartoon view of the interface, with the HA contact surface in orange. The Nb74 regions involved in binding, including framework region 1 (FR1) and complementarity-determining regions (CDR1, CDR2, and CDR3) of Nb74 are colored purple, green, yellow, and blue, respectively. Residues involved in hydrogen bonding are shown as sticks, with hydrogen bonds indicated by yellow dashed lines. Right: detailed view of the interface, with interacting HA residues labeled. (**I**) Structure of Nb74. Cartoon representation of Nb74, with antigen-binding regions colored as in panel H.

To elucidate the molecular basis of Nb74 recognition, we combined WB, escape mutant selection, and HDX-MS analyses. WB analysis was performed under denaturing conditions. Nb74 failed to recognize the denatured HA1 protein, indicating that Nb74 targets a conformation-dependent epitope (Fig. 7A and B).

To further define the antigenic region recognized by Nb74, escape mutants were generated by passaging virus in the presence of Nb74 in embryonated chicken eggs. Sequence analysis identified several amino acid substitutions in HA1, including T126K, K166M/E, S167L, and K169T/Q (H3 numbering; same hereafter; Fig. 7C, orange), suggesting that these residues contribute to Nb74 binding or viral escape. This supports that the identified escape residues, although discontinuous in sequence, are likely spatially clustered to form a conformational epitope. Notably, conservation analysis based on 436 HA1 amino acid sequences retrieved from the National Center for Biotechnology Information (NCBI) and Global Initiative on Sharing All Influenza Data (GISAID) database (as of June 2025) showed that these sites are generally conserved, with conservation rates of 93% (T126), 98% (K166), 100% (S167), and 72% (K169) (Fig. 7D), yielding an average conservation of 90.8%.

Peptide coverage and quality assessment demonstrated sequence coverage of HA1 with high-confidence peptides, supporting the robustness of the HDX-MS data set (Fig. S3A). Under these conditions, HDX-MS analysis revealed reduced deuterium uptake in multiple discrete regions of HA1 upon Nb74 binding, indicating a discontinuous but spatially clustered epitope. Specifically, five peptides exhibiting reduced deuterium uptake were identified, including peptides 58–66, 69–78, 115–125, 163–168, and 219–227 (Fig. 7E and F). Among these, peptide 115–125 (Fig. 7G, yellow) is adjacent to escape residue (126 T, Fig. 7G, orange), while peptide 163–168 (Fig. 7G, blue) contains two escape sites (166 K and 167 S, Fig. 7G, pink) and is also proximal to residue (K169, Fig. 7G, pink). These regions were, therefore, considered to represent the core epitope. In contrast, peptides 58–66 (Fig. 7G, red) and 69–78 (Fig. 7G, cyan) are spatially distant from the escape sites and were excluded, as they likely reflect indirect or distal conformational effects rather than direct nanobody binding. A slight spatial offset was observed between HDX-protected peptides and escape residues, reflecting the complementary nature of these approaches.

HDX-MS analysis revealed a pronounced change in deuterium uptake at residue 125 upon Nb74 binding (Fig. 7F), suggesting its potential involvement in the interaction interface. Based on this observation, a recombinant virus carrying the F125A substitution in the Rv1 background was generated using reverse genetics. However, attempts to rescue this mutant virus (F125A in Rv1, Table S1) were unsuccessful.

To predict the interaction interface between HA and Nb74, HA was modeled using SWISS-MODEL and Nb74 using AlphaFold3, followed by docking on the HADDOCK 2.4 server to generate the HA–Nb74 complex. The docking process was guided by residues identified from escape mutation and HDX-MS analyses, and the resulting models are shown in Fig. 7H and I. In the predicted complex, the putative antigenic epitope on HA is highlighted in orange and comprises residues G124, F125, T126, Y127, S128, G129, I130, N157, A160, P162, Q163, M164, T165, K166, S167, K169, T242, and T244, located on the back of the receptor-binding site (RBS) (right panel of Fig. 7H). Two potential hydrogen bonds were identified, one between V2 in FR1 of Nb74 and S128 of HA, and another between N110 in CDR3 and Q163 of HA (left panel of Fig. 7H). Those interacting residues on Nb74 are color-coded by region, with framework region 1 (FR1), complementarity-determining regions CDR1, CDR2, and CDR3 shown in purple, green, yellow, and blue, respectively (Fig. 7I). The predicted model suggests that Nb74 engages HA through a defined interface, including FR1 (Q1, V2, S25), CDR1 (V26, S27, P28, N29, and W30), CDR2 (I51), and CDR3 (G96, G97, S98, W99, Y100, 107S, 108E, 109Y, 110N, and 101Y). Collectively, these combined mutation, HDX-MS, and structural analyses demonstrate that Nb74 targets a highly conserved conformational epitope located on the lateral patch of HA.

## DISCUSSION

The interspecies transmission of H7 avian influenza viruses (AIVs) underscores their persistent capacity to drive zoonotic spillover events (4, 5, 40). Although reported human infections remain relatively infrequent compared with their widespread circulation in avian hosts, the diversity of H7 subtypes across geographic regions highlights a broader and underappreciated global risk (6). Notably, infections are often linked to poultry exposure, indicating that the threat posed by H7 viruses is not geographically restricted but instead reflects a dynamic and continually evolving interface between animal reservoirs and human populations (6). Within this global context, H7 AIVs have repeatedly caused outbreaks in poultry and sporadic but severe infections in humans, particularly in China. Although poultry vaccination has markedly reduced the number of human cases, the continuous evolution of RNA viruses sustains the risk of re-emergence and future epidemics (12, 41, 42). Despite these risks, no licensed human vaccine against H7 is available. Moreover, the virus retains several concerning features, including dual receptor affinity, relatively high case-fatality rates, and potential to acquire resistance to neuraminidase inhibitors. The lack of preexisting immunity to H7 viruses in human populations, combined with their high pathogenicity, highlights the urgent need for alternative therapeutic strategies. Although nanobodies possess promising antiviral properties, *in vivo* studies evaluating their efficacy against epidemic H7 influenza viruses are still scarce (25). Using Y2H screening, we isolated six nanobodies that exhibited hemagglutination HI activity and were specific for the HA1 subunit of hemagglutinin. Among them, Nb74 exhibited HI activity across a panel of H7 strains, including both vaccine strains and representative clinical isolates. *In vivo* administration conferred robust protection, as treated mice exhibited improved weight recovery, a reduced viral burden in the lungs, and alleviated pathology, highlighting its potential as a next-generation therapeutic for both the prevention and treatment of H7 virus infections.

Previous neutralizing antibodies, including 1H10, E10, 4H1E8, H7.HK1, and H7.HK2, conferred protection in animal models but required systemic injection at relatively high doses (21, 22, 25, 26). In comparison, Nb74 demonstrated superior neutralizing potency *in vitro*, with MN-IC_50_ values as low as 0.02 µg/mL, outperforming 1H10 (0.075 µg/mL) and E10 (2.34 µg/mL), potentially contributing to its efficacy at lower doses *in vivo*. In line with its potent *in vitro* activity, Nb74 also demonstrated robust *in vivo* efficacy when administered via the airway at relatively low doses. Prophylactic administration of 2 mg/kg Nb74 24 h before infection protected 80% of mice challenged with Rv2 and 100% with Rv1. Therapeutic administration at 4 mg/kg post-infection fully protected Rv1-infected animals and yielded 80% protection against Rv2. In the meantime, previously reported antibodies were primarily delivered via systemic routes, such as intravenous or intraperitoneal injection. For example, E10-Fc required intraperitoneal administration at 10 mg/kg prophylactically or 25 mg/kg therapeutically to achieve complete survival, while 4H1E8 required 20–30 mg/kg to confer 80%–100% protection. Similarly, H7.HK1 and H7.HK2 achieved 100% prophylactic efficacy and at least 80% therapeutic efficacy at 5 mg/kg. These differences in administration routes make direct comparisons with Nb74 difficult in the current study. The lower doses observed for Nb74 are, therefore, likely influenced by airway delivery, which may enhance local drug concentration at the site of infection while reducing systemic distribution or degradation (24, 43–46). Direct head-to-head comparisons under identical experimental conditions will be required to rigorously assess relative protective efficacy.

In addition, Nb74 neutralized Rv3 and multiple pre-2020 clinical isolates, supporting activity across diverse H7 strains. Together with its effective airway delivery, these findings highlight Nb74 as a promising antiviral candidate. Further optimization through multivalent or multi-specific nanobody engineering may improve binding avidity and neutralization potency, whereas nanobody cocktails could reduce viral escape (28, 47). Overall, this work highlights the potential of airway-delivered nanobodies as a minimally invasive therapeutic strategy for H7 avian influenza.

However, in the present study, Nb74 demonstrated variable neutralizing activity across different H7 strains, with no measurable MN activity observed against Rv4 and reduced HI activity against several recent clinical isolates. These findings indicate that Nb74 does not exhibit uniform potency across all tested viruses and suggest a more restricted spectrum of activity than initially anticipated. Such variability may reflect underlying antigenic differences among H7 viruses, particularly in regions contributing to the Nb74 epitope. Given that our HDX-MS and escape mutant analyses define a discontinuous and conformational epitope on HA1, even subtle amino acid substitutions introduced through antigenic drift could disrupt key interactions required for effective binding and neutralization. In addition, potential changes in glycosylation patterns may further influence epitope accessibility or alter the local structural environment, thereby reducing Nb74 efficacy against certain strains such as Rv4. These observations highlight the importance of epitope conservation in determining neutralization breadth and underscore the need for further investigation into the structural and evolutionary factors that govern Nb74 sensitivity across H7 lineages.

By integrating escape mutant profiling, HDX-MS analysis, and structural docking, we defined the epitope of Nb74 as a discontinuous yet spatially clustered region on HA1. Nevertheless, HDX-MS further identified multiple protected peptides that partially overlapped with escape residues and also extended to adjacent regions. While escape mutations identify residues that are functionally critical for nanobody binding, HDX-MS captures peptide-level changes in backbone exchange associated with local structural stabilization. Consequently, residues adjacent to escape sites may exhibit HDX protection, whereas some escape residues may not produce detectable HDX signals. The slight offset between HDX-protected peptides and escape residues underscores their complementarity, yielding a more comprehensive definition of the Nb74 epitope than either approach alone.

Several lateral patch-binding antibodies targeting H7 HA have been reported, including H7.HK1/2 and the nanobody E10 (25, 26). Based on our escape mutant profiling, HDX-MS analysis, and docking results indicate that the Nb74 epitope substantially overlaps with those of H7.HK1 and H7.HK2, with approximately half of the mapped residues shared between them. Notably, H7.HK1/2 bind to a region distal to the receptor-binding site (RBS) on the same protomer but are positioned closer to the RBS of a neighboring protomer and have been proposed to inhibit receptor engagement by inducing disorder in the 220 loop of the RBS (26). Consistent with the binding mode described for H7.HK1/2, HDX-MS analysis revealed that peptide 219–227 exhibited reduced deuterium uptake upon Nb74 binding (Fig. S3B). Concurrently, structural mapping showed that, although peptide 219–227 is distant from escape-associated residues within a single HA protomer, this peptide lies in close proximity to those residues on an adjacent protomer in the trimeric structure (Fig. S3C). Based on these observations, it is plausible that Nb74 recognizes a conserved quaternary epitope spanning neighboring HA subunits. This interaction may alter the local conformation of the HA trimer or sterically hinder the receptor-binding site, thereby reducing receptor engagement without directly occupying the RBS. This may explain its ability to inhibit viral attachment to host cells. Also, the recognition of a quaternary epitope spanning adjacent HA1 subunits likely explains why Nb74 potently neutralizes virus despite only moderate SPR-measured affinity for monomeric HA1. HDX-MS analyses suggest involvement of the 219–227 region; however, as these data are resolved at the peptide level and may also reflect contributions from adjacent residues, the precise interacting residues remain unclear. Future studies will employ high-resolution structural approaches, followed by targeted mutagenesis to define the interaction interface.

Previous nanobodies identified through phage display or yeast display have predominantly been reported to recognize relatively defined or linear epitopes (25, 38), providing a broader framework for understanding nanobody-HA interactions. In contrast, our study integrates functional assays with epitope mapping to provide mechanistic insight into Nb74-mediated neutralization. Unlike the previously reported nanobody E10, which was isolated by phage display and primarily recognizes a linear epitope within residues 166–186 based on phage display and escape mutant analyses (25), Nb74 was obtained through Y2H screening using native-like HA. It binds a discontinuous, spatially distributed epitope in the lateral patch, as revealed by HDX-MS and escape mutant profiling. Functionally, both Nb74 and E10 inhibit viral attachment to host cells. Despite this shared outcome, their distinct epitope architectures indicate that different regions of HA can mediate attachment inhibition. However, for Nb74, this effect may stem from conformational changes or steric constraints that reduce receptor binding, affecting the RBS directly or its accessibility on an adjacent HA protomer. In addition, the *in vivo* efficacy of E10 has been evaluated using an Fc-fused format administered via intraperitoneal injection, whereas Nb74 was tested via intratracheal delivery, a route more directly relevant to respiratory infection. In this context, Nb74 was not fused to an Fc domain, as its small molecular size is advantageous for local diffusion and tissue penetration in the lung following intratracheal administration. Nb74 conferred robust protection under these conditions, supporting its potential for both prophylactic and therapeutic applications. However, given the differences in molecular format and administration routes, direct comparisons of efficacy between Nb74 and E10 are not possible in the current study. Taken together, this work provides insights into H7-specific antibody responses and supports rational vaccine design.

Despite these findings, several limitations should be acknowledged. First, the epitope of Nb74 was defined based on HDX-MS, escape mutant profiling, and computational docking, rather than high-resolution structural determination. Although these complementary approaches provide convergent evidence, atomic-level details of the Nb74-HA interaction remain to be resolved. And, the discrepancy between measured affinity and neutralizing potency is not fully captured by current study. Future studies could address these limitations by determining the high-resolution structure of the Nb74-HA complex, for example, using co-crystallization or cryo-EM, to precisely define its binding mode and quaternary interactions. In addition, analyses using trimeric or virion-associated HA could provide a more accurate assessment of its binding properties in a native-like context. Such efforts will not only refine our understanding of the neutralization mechanism but also inform the rational design of protective antibodies and vaccines. In parallel, engineering strategies, including affinity maturation, CDR walking, and site-directed mutagenesis, could be applied to strengthen nanobody binding and broaden neutralization (48–51). These methods have proven effective in enhancing viral nanobodies, including those against SARS-CoV-2, and hold promise for adapting Nb74 to counter emerging H7 variants (52). Also, we did not systematically evaluate potential pulmonary toxicity, inflammatory responses, or the induction of anti-nanobody antibodies following intratracheal administration. These aspects are important for further preclinical development, especially considering the non-murine origin of Nb74. Future studies will focus on comprehensive assessments of pulmonary safety, immunogenicity, and host immune responses to better support the translational potential of this nanobody.

## MATERIALS AND METHODS

### Cells, viruses, and proteins

293T and MDCK cells were propagated and maintained in Dulbecco’s modified Eagle’s medium (DMEM; Gibco, MA, USA) supplemented with 10% fetal bovine serum (FBS; Gibco) at 37°C under 5% CO_2_. 293T cells were used for recombinant virus rescue, and MDCK cells were used for virus propagation, determination of tissue culture infectious dose (TCID_50_), and neutralization assays. Sf9 cells were cultivated in Grace’s insect medium at 28°C.

Recombinant H7 influenza viruses were generated using an eight-plasmid reverse genetics system (53). Rv1, Rv2, Rv3, and Rv4 harbored seven internal segments from A/Puerto Rico/8/1934 (H1N1) and HA segments from A/pigeon/Shanghai/S1069/2013 (H7N9), A/chicken/Guangxi/SD098/2017 (H7N9), A/chicken/Inner Mongolia/SD010/2019 (H7N9), and A/chicken/Yunnan/SD024/2021 (H7N9), respectively, which correspond to the HA origins of the vaccine strains H7-Re1 through H7-Re4 (Table S2). For epitope mapping, single-site mutations were introduced into Rv1 HA, replacing residues with F125A (H3 numbering, Table S2). Eight internal gene segments of the influenza virus were separately introduced into pDZ at *BsmB*I restriction sites and co-transfected into 293T cells using Lipofectamine 2000 (Invitrogen, USA) for virus rescue. Viral recovery was verified by a hemagglutination assay and full-genome sequencing (primers LHN-F and LHN-R; all primers listed in Table S4), and titers were determined using a conventional TCID_50_ assay as described previously (54).

The recombinant HA1 protein (residues 11–329, H3 numbering, Table S2) corresponded to the viral sequence, and its amino acid sequence is provided in Table S1. The cDNA of the recombinant HA1 domain was cloned and inserted into the baculovirus shuttle vector pFastBac Dual, coupled with the gp67 secretion signal peptide and C-terminal 8 × His tag. Soluble HA1 was expressed in Sf9 cells using the Bac-to-Bac Baculovirus Expression System (Invitrogen). Primers, amino acid sequences, plasmids, and strains are listed in Tables S1, S5 and S6. After purification from the culture supernatant (Genscript, China), the HA1 protein concentration was measured by a BCA assay (Vazyme, China) and used for ELISA and surface plasmon resonance (SPR) binding studies.

### Yeast two-hybrid library construction

A healthy 2-year-old male Bactrian camel received five immunizations with the inactivated H7-Re1 vaccine (Harbin Weike Biotechnology, China) at 3-week intervals. One month after the final booster, peripheral blood mononuclear cells (PBMCs) were isolated from 200 mL of fresh blood, and total RNA was extracted for VHH amplification. VHH fragments were generated by nested PCR (primers in Table S4), ligated into linearized pGADT7-Rec, and expressed as fusions with the GAL4 activation domain (39). The generated plasmids were first prepared for transformation. They were introduced into the yeast strain Y187 using lithium acetate and the Yeastmaker Yeast Transformation System 2 (Clontech, CA, USA). The resulting prey library was stored at −80°C. Library quality was assessed by plating gradient dilutions on SD/-Leu agar (synthetic dropout medium lacking leucine, *n* = 2 per dilution) for colony counting. Insertion diversity was evaluated by PCR and sequencing of 25 randomly selected colonies using the primers GAL4AD-F and 3AD-R (Table S4).

### Nanobody screening by Y2H assay

The bait strain Y2HGold harboring pGBKT7-HA1 was constructed by cloning Rv1-HA1 into pGBKT7 (primers in Table S4) fused with the GAL4 binding domain. Self-activation was assessed on SD/–Trp/X-α-Gal and SD/–Trp/X-α-Gal/AbA plates, and toxicity was excluded by comparing colony growth with that of pGBKT7-empty on SD/–Trp plates. For screening, Y2HGold carrying pGBKT7-HA1 was mated with the Y187 nanobody library following the Matchmaker Gold protocol (Clontech). Candidate colonies were first selected on DDO/X/A (SD/–Leu/–Trp supplemented with 40 µg/mL X-alpha (α)-Gal and 200 ng/mL aureobasidin A; Takara Bio, USA) and subsequently confirmed on QDO/X/A (SD/–Leu/–Trp/–Ade/–His with the same supplements) plates. Next, candidate clones were first validated by PCR and sequencing, followed by co-transformation with pGBKT7-HA1 or the empty pGBKT7 vector to distinguish true interactions from false positives. Controls comprising pGBKT7-HA1 + pGADT7-empty, pGBKT7−53 + pGADT7T, and pGBKT7-Lam + pGADT7T were included. VHH sequences derived from positive colonies were aligned using MUSCLE, and a phylogenetic tree was constructed using the maximal likelihood method in MEGA11.

### Nanobody production

VHH genes (Table S1) with a C-terminal 6-His or Twin-Strep-tag were inserted into the pPICZαA vector using *EcoR*I and *Xba*I sites and synthesized by General Biosystems (Anhui, China) Co., Ltd. The constructs were individually introduced into *P. pastoris* X33 (Mut^+^/His^+^) and cultured in yeast extract peptone dextrose medium. Following the instructions of EasySelect Pichia Expression Kit, the recombinant strains were confirmed by PCR (primers 5α-Factor-F and 3AOX1-R in Table S4) and induced to express nanobodies in a buffered minimal methanol medium at 28°C–30°C (55). The molecular weight and purity of the nanobodies were characterized by SDS-PAGE.

### Hemagglutination inhibition assay

Virus hemagglutination units (HAU) were measured using a standard hemagglutination assay (56). For HI testing, a volume equivalent of 4 HAU of virus was added to 96-well V-bottom plates containing twofold serially diluted nanobodies. The plates were shaken gently, and an equal volume of 1% chicken red blood cells (RBCs) was added after 30 min. Hemagglutination was scored by visual examination. The IC_50_ value was reported as the nanobody concentration that inhibited the hemagglutination of 50% of RBCs.

### Virus microneutralization assay

As described previously (21, 57), an equal volume of 100 TCID_50_ virus was stained with a twofold serial dilution of the nanobodies for one hour. The virus plus VHH mixture was added to the MDCK cells at 37°C and removed after 2 h. The cells were subsequently washed and cultured in maintenance medium (2% BSA-DMEM supplemented with 2 µg/mL TPCK-treated trypsin, hereafter) for 48 h. Cells were fixed with paraformaldehyde, permeabilized using 0.2% Triton X-100, and probed with a mouse monoclonal antibody (mAb) against influenza A nucleoprotein (NP, GenTex, USA) followed by anti-mouse IgG FITC (Sangon, Shanghai, China). The IC_50_ value represented the nanobody concentration that inhibited 50% of viral reproduction. Positive controls (virus-infected cells) were included at the same time.

### Binding assays

The binding activity of the nanobodies to the H7 HA1 protein was evaluated by ELISA. Plates were incubated with 1 µg of baculovirus-derived H7 HA1 protein in carbonate-bicarbonate buffer (pH 9.6) and blocked with 3% BSA. Purified serially diluted Twin-Strep-tagged nanobodies were added at a starting concentration of 5 µg/mL and incubated for 1 h at room temperature. Bound nanobodies were detected using an anti-Strep tag II rabbit antibody (1:2,000; GenScript, Nanjing, China) followed by horseradish peroxidase-conjugated goat anti-rabbit IgG (1:5,000; Sangon), each for 1 h. Then, 3,3′,5,5′-tetramethylbenzidine (Thermo, MA, USA) was added, and the reaction was halted with 2 M H_2_SO_4_. Plates were rinsed with PBST between steps, with Nb06 used as a negative control. The absorbance at 450 nm was measured using a Bio-Tek Epoch 2 reader. The EC_50_ values were calculated using four parameters for nonlinear regression fitting in GraphPad Prism 9.

The interaction between the nanobodies and recombinant HA1 proteins was characterized via SPR on a Biacore 8K (GE Healthcare, USA) to determine the binding kinetics and affinities (58). The ligand HA1 (10 µg/mL) in PBS was immobilized on flow cell 2 of a saturated Ni-NTA sensor chip (His Capture Kit; GE Healthcare) at 10 µL/min for 3 min, while flow cell 1 without an immobilized ligand served as the reference surface. Association (*K*_a_, M^−1^s^−1^) and dissociation (*K*_d_, s^−1^) values were monitored when the Twin-Strep-tagged concentration gradient started at 2,000 nM and underwent successive twofold dilution. The analyte was passed through at 30 µL/min for a contact phase of 120 s, followed by a 600 s dissociation phase, which was recorded in both flow cells at 25°C with a multicycle method. Responses from reference flow cells were subtracted to establish the response units linked to specific binding. *K*_a_, *K*_d_, and the equilibrium dissociation constant (*K*_D_) were calculated by global fitting with a 1:1 (Langmuir) binding model using BIA evaluation software (GE Healthcare). The data were exported and visualized in GraphPad Prism.

### Mechanistic assays

A series of mechanistic assays were conducted to elucidate the antiviral activity of Nb74. Entry inhibition was identified by a cell-based entry inhibition assay with minor modifications (59, 60). Rv1 virus was preincubated with Nb74 (5 or 20 µg/mL) and then applied to MDCK cells for 1 h at 4°C to allow viral binding while preventing internalization. After incubation, the cells were washed three times with cold phosphate-buffered saline (PBS) to remove unbound virus. For attachment analysis, cells were fixed immediately and subjected to downstream detection. Alternatively, to evaluate subsequent infection, cells were shifted to 37°C and incubated in nanobody-free medium for 16–18 h prior to analysis. The cells were fixed with 80% acetone, permeabilized using 0.2% Triton X-100, and incubated with 3% BSA. The plates were subsequently rinsed, and the infected cells were quantified using a mouse anti-NP mAb and anti-mouse IgG FITC secondary antibody, after which the nuclei were counterstained with DAPI. Antibody binding and infection levels were observed by immunofluorescence microscopy.

Syncytium formation was analyzed to examine the Nb74-mediated inhibition of cell-cell fusion (21, 59). Briefly, MDCK monolayers were infected with Rv1 at an MOI of 0.5 for 24 h. The cells were treated with 0, 5, or 20 µg/mL Nb74 in DMEM for 1 h, followed by exposure to citric acid (pH 5.5) at 37°C for 10 min and thorough washing. The cells were incubated with maintenance medium for 3 h, fixed with paraformaldehyde, and stained with Giemsa. The mock group consisted of uninfected MDCK cells subjected to the same treatment. The relative syncytial area of syncytium formation was observed by light microscopy and evaluated by three people.

Egress inhibition was examined by evaluating viral release (59, 60). MDCK cells were exposed to Rv1 at an MOI of 2 for 4 h, rinsed to remove unbound virus, and maintained in medium supplemented with varying concentrations of Nb74 for 18–22 h. Both the supernatants and the cell lysates were collected and analyzed by WB to quantify the virions discharged into the supernatant. The samples were run on a 12% polyacrylamide gel and subsequently blotted onto a 0.45-μm polyvinylidene difluoride (PVDF) membrane. After blocking with 10% nonfat milk, the membrane was incubated with anti-NP mAb and anti-β-actin antibody (Proteintech, Wuhan, China), followed by appropriate HRP-conjugated secondary antibody (Sangon). The membranes were rinsed with PBST between each step. Enhanced chemiluminescence (Yeason, Shanghai, China) was used to observe the immunoreactive bands.

### Prophylactic and therapeutic protective activities in mice

Six-week-old female SPF BALB/c mice (SPF, Beijing, China) were anesthetized with 3% inhaled isoflurane and intranasally challenged with 10 MLD_50_ of Rv1, Rv2, or Rv3. Nb74 or PBS was intratracheally administered with a microspray aerosolizer (Yuyan Bio, Shanghai, China) under anesthesia induced by intraperitoneal ketamine/xylazine injection. To determine the prophylactic efficacy, mice (*n* = 8) were treated with 2 or 3 mg/kg Nb74 or an equivalent volume of PBS 24 h before viral challenge. To assess the therapeutic effects, 4 or 8 mg/kg Nb74 or PBS was administered to the sedated mice 24 h post-challenge. Within each group, five mice were monitored longitudinally for survival and body weight over a 14-day period, whereas the remaining three mice were designated for endpoint analyses, including lung viral load determination and histopathological examination at day 5 post-infection. Mice were humanely euthanized and marked as dead when body weight loss exceeded 25% relative to the challenge day.

All animal experiments were conducted in accordance with institutional guidelines and approved by the relevant Animal Care and Use Committee, and all procedures involving live viruses were performed in appropriate biosafety containment facilities.

### Histological and viral titration analysis

To further characterize the protective effect, lungs from three mice per group were collected at day 5 post-challenge (61). The left lungs were removed and preserved in 10% neutral buffered formalin for 2 days. After dehydration and fixation in paraffin, the tissues were sectioned (5–8 µm), deparaffinized and stained with hematoxylin and eosin (HE). Common lung injury was assessed by the infiltration of inflammatory cells, mainly lymphocytes and macrophages; congestion and expansion of the alveolar septa with inflammatory cell infiltration; inflammatory cells surrounding the blood vessels; and serous exudate within the alveolar spaces. Lesion severity was assessed on the basis of the percentage of tissue affected: 0 for no lesions, 1 for minimal (1%–20%), 2 for moderate (21%–50%), and 3 for severe (51%–100%).

For viral titration, half of the lung tissues were homogenized in PBS and centrifuged. The supernatants were serially diluted 10-fold and inoculated onto confluent MDCK cells in 96-well plates. After 1 h incubation, the inoculum was removed, and the cells were washed three times and cultured for 2 days in maintenance medium. Viral titers were determined as the 50% tissue culture infectious dose (TCID_50_) and calculated using the Reed-Muench method (62).

### Epitope type analysis by Western blot

WB analysis was performed to characterize the epitope type recognized by Nb74 on HA1 (21, 63). Purified Rv1-HA1 proteins and Twin-Strep-tagged Nb74 (used as a control) were resolved by 12% SDS-PAGE and transferred onto 0.45-µm PVDF membranes. The membranes were blocked with 5% nonfat milk, incubated with Twin-Strep-tagged Nb74, and probed with a rabbit anti-Strep-tag II antibody. A parallel blot containing HA1 alone was detected with a mouse anti-His-tag mAb. After washing with PBST, HRP-conjugated secondary antibodies (goat anti-rabbit or anti-mouse IgG) were applied. And the signal was developed using enhanced chemiluminescence (ECL) substrate (Yeason, China).

### Selection of escape mutants

Escape mutant selection was performed to determine the epitope targeted by Nb74, following the procedure previously described by He et al. (57). In brief, recombinant H7-Rv1 virus was mixed with excess Nb74 for 1 h before inoculation into 10-day-old embryonated chicken eggs. The inoculated eggs were maintained at 37°C for 48 h to allow viral replication. Progeny viruses were subsequently collected and subjected to limiting-dilution cloning in embryonated eggs to isolate escape variants, which were further plaque purified. The HA genes of the selected viruses were sequenced, and the mutations were determined by comparison with the parental virus sequence.

### Hydrogen-deuterium exchange mass spectrometry analysis

Prior to HDX analysis, peptide mapping of HA1 was performed using the undeuterated protein. HA1 was digested under the same conditions and liquid chromatography (LC) gradient as described for HDX experiments. The resulting peptides were analyzed by MSE acquisition on a Synapt G2-Si mass spectrometer (Waters), employing a collision energy ramp ranging from 20 to 30 kV. Sodium iodide was used for instrument calibration, and Leucine Enkephalin served as the lock-mass standard to ensure mass accuracy. The MSE data sets were processed using ProteinLynx Global Server (PLGS) version 3.0 (Waters). And peptides were further filtered in DynamX 3.0 (Waters).

For deuterium exchange experiments, 21 pmol HA1 were incubated alone or with 7 pmol of Nb74 (molar ratio 3:1) in a total reaction volume of 1 µL per time point. Following equilibration, deuterium labeling was initiated by a fivefold dilution into deuterated PBS (pH 7.35), yielding final labeling time points of 0.1, 1, 10, and 60 min at 25°C. Exchange reactions were quenched by adding ice-cold quenching buffer at a ratio of 1:3 (vol/vol), consisting of 100 mM phosphate buffer supplemented with 4 M urea and 0.5 M TCEP (66%, vol/vol), combined with labeling buffer (33%, vol/vol), resulting in a final pH of 2.3. Samples were incubated on ice for 30 s and immediately snap-frozen in liquid nitrogen. All conditions were performed in duplicate, and samples were stored at −80°C prior to analysis.

For LC-MS analysis, samples were rapidly thawed and injected into an Acquity UPLC M-Class system equipped with HDX technology (Waters). Online proteolysis was carried out at 20°C using a dual protease column (Pepsin-Type XIII, 1:1; NovaBioAssays). Peptides were subsequently trapped and desalted for 3 min at 200 µL/min using Solvent A (0.23% formic acid in water, pH 2.5) at 0°C on an Acquity BEH C18 VanGuard pre-column (1.7 µL, 2.1 mm × 5 mm; Waters).

Peptide separation was achieved on an Acquity UPLC BEH C18 analytical column (1.7 µL, 2.1 mm × 100 mm, Waters) using a linear gradient of 8%–40% Solvent B (0.23% formic acid in acetonitrile) at a flow rate of 40 µL/min and maintained at 0°C. Eluted peptides were ionized via electrospray ionization in positive mode and analyzed by mass spectrometry with ion mobility separation. To minimize peptide carryover, the protease column was thoroughly cleaned between runs using 1.5 M guanidine hydrochloride in 100 mM phosphate buffer (pH 2.5). Additionally, a saw-tooth gradient was applied to wash the chromatographic system.

### HA sequence analysis

H7 HA amino acid sequences were collected from the GISAID (https://www.gisaid.org/) database and the GenBank Influenza Virus Resource (https://www.ncbi.nlm.nih.gov/genomes/FLU/Database/nph-select.cgi?go_database). Multiple sequence alignment was processed and analyzed using MEGA11. Redundant sequences were excluded, and amino acid composition analysis of HA1 was conducted using Bioinformatics Aider. All HA residue positions mentioned in this study are numbered according to the H3 numbering scheme.

### Structural modeling

A structural model of the nanobody was predicted with AlphaFold 3 (https://alphafoldserver.com/), while the Re1-HA structure was constructed via SWISS-MODEL (https://swissmodel.expasy.org/). Docking simulations of Nb74 with Re1-HA were carried out using the HADDOCK 2.4 server (https://wenmr.science.uu.nl/haddock2.4/), followed by interface analysis using PDBePISA (https://www.ebi.ac.uk/pdbe/pisa/). Structural figures were prepared with PyMOL v2.5.5 (Schrödinger).

### Statistical analysis

Data analysis was performed using GraphPad Prism 9.0 software. Survival curves were compared using Kaplan-Meier survival analysis with a log-rank test. One-way or two-way analysis of variance (ANOVA) is described in the figure legends. A *P* value of <0.05 was considered to indicate statistical significance. Asterisks represent the following levels of significance: ns, not significant; *P < 0.05, **P < 0.01, ***P < 0.001, ****P < 0.0001.

## Data Availability

All data generated or analyzed during this study are included in this published article and its supplemental material.

## SUPPLEMENTAL MATERIAL

Supplementary data jvi.00563-26-s0001.docx Tables S1 to S6; Fig. S1 to S3.

ASM does not own the copyrights to Supplemental Material that may be linked to, or accessed through, an article. The authors have granted ASM a non-exclusive, world-wide license to publish the Supplemental Material files. Please contact the corresponding author directly for reuse.
