# Supplementary material for: A neutralizing nanobody targeting a conserved lateral patch on HA1 confers protection against multiple H7 avian influenza viruses
Source: J Virol. 2026 Jun 11;100(7):e00563-26. doi: 10.1128/jvi.00563-26 (PMC13386974; doi:10.1128/jvi.00563-26)
Supplement: Supplementary data — Tables S1 to S6; Fig. S1 to S3. [file jvi.00563-26-s0001.docx]

**Supplementary materials for**

**A neutralizing nanobody targeting a conserved lateral patch on HA1 confers protection against multiple H7 avian influenza viruses**

**Authors**

Siqi Xu^a^, Qinying Zhang^a^, Xueer Xie^a^, Mengruo Zhou^a^, Yunxia Chen^a^, Yutong Liu^a^, Chenying Luo^b^, Qi Zhang^a^, Han Zheng^a^, Saixiang Feng^a^ *, Ming Liao^a,c,d^ *

^a^ College of Veterinary Medicine, South China Agricultural University, Guangzhou, 510642, China

^b^ College of Life Sciences, South China Agricultural University, Guangzhou, 510642, China

^c^ Institute of Animal Health, Guangdong Academy of Agricultural Sciences, Guangzhou, 510640, China

^d^ Zhongkai University of Agricultural and Engineering, Guangzhou, 510550, China

***Correspondence**

Saixiang Feng: +86-20-85280718; fengsx@scau.edu.cn

Ming Liao: +86-20-89003055; mliao@scau.edu.cn

**Contents**

**Table S1. Amino sequences of nanobodies and HA1 protein used in this study.**

**Table S2.** **Recombinant virus strains and HA1 proteins used in this study.**

**Table S3. Summary of neutralization potency and breadth of nanobodies across influenza virus strains viruses.**

**Table S4. Primers used in this study.**

**Table S5. Plasmid used in this study.**

**Table S6. Bacterial and yeast strains used in this study.**

**Figure S1. Comprehensive quality assessment of the yeast two-hybrid nanobody library and validation of bait functionality and recombinant protein expression.**

**Figure S2. Raw SPR sensorgrams of Nb74 binding to Rv1-Rv4 HA1 proteins.**

**Figure S3. Structural interpretation of Nb74 epitope suggests a potential quaternary binding mode.**

**Table S1. Amino sequences of nanobodies and HA1 protein used in this study.**

| **Name** | **Animo sequence** |
| --- | --- |
| Nb04 | QVQLVESGGGLVQPGGSLRLSCAASGFTFSSYAMSWVRQAPGKGLEWVAAIFSGGGREYYADSVKGRFTISRDNAKNTAYLQLSSLKTEDMAMYYCAKPKIADRWYELSALDAWGQGTQVTVSS |
| Nb05 | QVQLVESGGGLVQPGGSLTLSCVASGFPLSTHDMRWVRQAPGTGFEWVSYINSQGGRTYYADSVKGRFTISRDNAKNTVYLQLNNLKTEDMAMYYCWTDFGSWGQGTQVTVSS |
| Nb36 | QVQVGECGGGSVQGGGSLMLSCAASGYTSSRCSMGWYRQAPGVERELVVNIISDGSIWYAESVKGRFTAFQDNAKNILCLRMNSLRPEDTAMYYCNTATWSGGSCDSGGHGRYSLWGQGTQVTVSS |
| Nb66 | QVQLVESGGGSAQAGGSLRLSCAASGYIANSCSMGWYRQAPGKERELVSIIIEGSRIIYLDSVKGRFTISQDNAKSAMYLEMNSLKPEDTAIYYCNICVWNGGSCDSGGRSRYNYWGQGTQVTVSS |
| Nb67 | QVQLVESGGGSVQVGGSLTLSCVASGYTDNYCSMGWYRQAPGQPRELVSAIISTGRTVYAESAKGRFTIFQNNAKNTVYLQMNSLRSEDTAMYYCNIAVWNGGSCDSGGRGRYNYWGQGTQVTVSS |
| Nb74 | QVQLVESGGDSVQAGGSLRLSCAASVSPNWCMGWFRQAPGKEREVISAIDIDGSTHYAGAVKGRFTISQDKAKNAVYLQMDGLKPEDTAMYYCAAGGSWYCPVLTISEYNYWGQGTQVTVSS |
| Nb77 | QVQLVESGGGLVQPGESLRLSCVASGFTFSNYDMMWVRQAPGKGLEWVSGISGGGGMRNYADSVKGRFTISRDNPKNTVYLRLNSLKTEDMAMYYCARGYAGTWYMHWGQGTQVTVSS |
| Nb83 | QVQLVESGGGSVQAGGSLMLSCAASGYTSSRCSMGWYRQAPGLERELVVNIISDGSIWYAESVKGRFTAFQDNAKNILYLQMNSLRPEDTAMYYCNTATWNGGSCDSGGHGRYNLWGQGTQVTVSS |
| Nb108 | QVQLVESGGGSVQAGGSLRLSCAASEYIDRTCSLGWYRQAPGKEREMVAVVIEGGRTVYLDSVKGRFMISQFNTKNTVYLQMNSLKPEDTAMYFCNIVIWNGGSCDSGGHSRYNYWGQGTQVTVSS |
| Nb110 | QVQLVESGGGSVQGGGSLRLSCAHSEYSGRVCMAWFRQAPGKEREAVAAISTRTGGTYEYYADSVKGRFSISQDNANMFLQMNSLKPEDTAMYYCASPGPWVHCFSGTWASRGQYNYWGQGTQVTVSS |
| Nb111 | QVQLVESGGGLVQPGGSLRLSCTASGNTGSRFCMGWLREAPGKEREVVAAIDIDGSTHYADSVKGRFTISQDNVENTLTLEMNSLKPEDTARYYCAAGGSWYCPRLTTTEYNYWGPGTQVTVSS |
| Nb120 | QVQLVESGGGSVQTGGSLTLSCEASRAPDRTEYMGWFRQAPGKEREGVAGIESDGSTVYANSVKGRFTISRDGNTLRLQMNTLQPADTGMYYCARDSTCNRLSMSAMSACFHFGDWGQGTQVTVSS |
| Nb122 | QVQLVESGGGSVQAGESLRLSCTVSGFNFDDSDMGWYRQGPGNQCEVVSSITTDGSTYYPDSVKGRFTISQDRGKDTLYLQMNSLKVEDTGVYYCAAPVLVTNAGTCAALGLLWEKLDEGFWGQGTQVTVSS |
| Nb127 | QVQLVESGGGLVQPGGSLRLSCVVSSHIYKPYCMAWFRQAPGKEREGIASIDGDGTVVHAESVKGRFTTSQDNTKTTLYLQMNSLKPEDTAMYYCAADPDSPTCSPNGGSWYIDWQFGYRGQGTQVTVSS |
| Nb131 | QVQLVESGGRSVQPGETLTLSCTASGFTFENSNMGWFRQAPGNGCELVSSINSDGSTYYVDSVKGRFTISQDNAKITVYLQMNSLKPEDTAVYHCTPRGLYSDYGLGCWGQGTQVTVSS |
| Nb138 | QVQLVESGGGLVQPGGSLRLSCAGSGFTFSNYWMYWVRQAPGKGLEWVSTSAGGSTYYGDSVKGRFTISRDNAKNTVYLQMDSLKPEDTAIYYCVRDLGGLATVTIGYWGQGTQVTVSS |
| Nb06 | QVQLVESGGGLVQPGGSLRLSCAASGFTFSNNWMHWVRQAPGKGLEWVSSINTGDGSTNSADSVKDRFTISRDNAKNTLYLQLNSLKTEDTAMYYCAKAAASWHIIHSWGQGTQVTVSS |
| Rv1-HA1 | DKICLGHHAVSNGTKVNTLTERGVEVVNATETVERTNIPRICSKGKRTVDLGQCGLLGTITGPPQCDQFLEFSADLIIERREGSDVCYPGKFVNEEALRQILRESGGIDKEAMGFTYSGIRTNGATSACRRSGSSFYAEMKWLLSNTDNAAFPQMTKSYKNTRKSPALIVWGIHHSVSTAEQTKLYGSGNKLVTVGSSNYQQSFVPSPGARPQVNGLSGRIDFHWLMLNPNDTITFSFNGAFIAPDRASFLRGKSMGIQSGVQVDANCEGDCYHSGGTIISNLPFQNIDSRAVGKCPRYVKQRSLLLATGMKNVPEIPKGR |
| Rv2-HA1 | DKICLGHHAVSNGTKVNTLTEKGVEVVNATETVERTNTPRICSKGKRTVDLGQCGLLGTITGPPQCDQFLEFSADLIIERREGSDVCYPGKFVNEEALRQILRESGGIDKEPMGFTYNGIRTNGVTSACRRSGSSFYAEMKWLLSNTDNAAFPQMTKSYKNTRESPAIVVWGIHHSVSTAEQTKLYGSGNKLVTVGSSNYQQSFVPSPGARPQVNGQSGRIDFHWLILNPNDTVTFSFNGAFIAPDRASFLRGKSMGIQSGVQVDANCEGDCYHSGGTIISNLPFQNIDSRAVGKCPRYVKQRSLLLATGMKNVPEVPKRKRTAR |
| Rv3-HA1 | DKICLGHHSVSNGTKVNTLTEKGVEVVNATETVERTNTPRICSKGKRTVDLGQCGLLGTITGPPQCDQFLKFSADLIVERREGSDVCYPGKFVNEEALRQILRESGGIDKEPMGFKYNGIRTNGTTSACRRSGPSFYAEMKWLLSNTDNATFPQMTKSYKNTRESPAIVVWGIHHSVSTAEQTKLYGSGNKLVTVGSSNYQQSFVPSPGARPQVNGQSGRIDFHWLILNPNDTVTFSFNGAFIAPDRASFLRGKSMGIQSGVQVDANCEGDCYHSGGTIISNLPFQNIDSRAVGKCPRYVRQRSLLLATGMKNVPEVPKRKRTAR |
| Rv4-HA1 | DKICLGHHSVSNGTKVNTLTEKGVEVVNATETVERTNTPRICSKGKKTVDLGRCGLLGTITGPPQCDQFLKFSADLIVERREGSDVCYPGKFVNEEALRQILRESGGIDKESMGFKYDGIRTNGTTSACMRSRSSFYAEMKWLLSNTDNATFPQMTKSYKNTRESPAIVVWGIHHSVSTAEQTKLYGSGDKLVTVESSNYQQSFVPSPGARPKVNGQSGRIDFHWLILNPNDTVTFSFNGAFIAPDRASFLRGKSMGIQSGVQVDANCEGDCYHSGGTIISNLPFQNVDSRAVGKCPRYVRQRSLLLATGMKNVPEVPKRKRTAR |

**Table S2. Recombinant virus strains and HA1 proteins used in this study.**

| **Sample** | **HA donor virus** | **HA Source** | **Accession number** | **Source** |
| --- | --- | --- | --- | --- |
| Rv1 | A/pigeon/Shanghai/S1069/2013(H7N9) | H7-Re1 | EPI440701 | This study |
| Rv2 | A/chicken/Guangxi/SD098/2017(H7N9) | H7-Re2 | EPI1555640 | This study |
| Rv3 | A/chicken/Inner Mongolia/SD010/2019(H7N9) | H7-Re3 | EPI1853891 | This study |
| Rv4 | A/chicken/Yunnan/SD024/2021(H7N9) | H7-Re4 | EPI3220096 | This study |
| F135A in Rv1 | A/pigeon/Shanghai/S1069/2013(H7N9) | H7-Re1 | EPI440701 | / |
| Rv1-HA1 | A/pigeon/Shanghai/S1069/2013(H7N9) | H7-Re1 | EPI440701 | This study |
| Rv2-HA1 | A/chicken/Guangxi/SD098/2017(H7N9) | H7-Re2 | EPI1555640 | This study |
| Rv3-HA1 | A/chicken/Inner Mongolia/SD010/2019(H7N9) | H7-Re3 | EPI1853891 | This study |
| Rv4-HA1 | A/chicken/Yunnan/SD024/2021(H7N9) | H7-Re4 | EPI3220096 | This study |

The accession number obtained from Global initiative on sharing all influenza data (GSAID).

**Table S3. Summary of neutralization potency and breadth of nanobodies across influenza virus strains viruses.**

| **Nanobody** | **Virus** | **HI-IC_50_** | **MN-IC_50_** |
| --- | --- | --- | --- |
| Nb66 | Rv1 | 19.79 ± 3.96 | 3.17 ± 0.79 |
|  | Rv2 | >50 | >50 |
|  | Rv3 | >50 | >50 |
|  | Rv4 | >50 | >50 |
| Nb67 | Rv1 | 21.35 ± 4.27 | 3.42 ± 0.85 |
|  | Rv2 | >50 | >50 |
|  | Rv3 | >50 | >50 |
|  | Rv4 | >50 | >50 |
| Nb74 | Rv1 | 0.23 ± 0.06 | 0.02 ± 0.01 |
|  | Rv2 | 0.57 ± 0.11 | 0.06 ± 0.01 |
|  | Rv3 | 3.65 ± 0.91 | 1.09 ± 0.00 |
|  | Rv4 | 43.75 ± 0.00 | >50 |
| Nb83 | Rv1 | 32.29 ± 6.46 | 10.33 ± 2.58 |
|  | Rv2 | >50 | >50 |
|  | Rv3 | >50 | >50 |
|  | Rv4 | >50 | >50 |
| Nb108 | Rv1 | 18.23 ± 3.65 | 0.73 ± 0.18 |
|  | Rv2 | >50 | >50 |
|  | Rv3 | >50 | >50 |
|  | Rv4 | >50 | >50 |
| Nb111 | Rv1 | 28.65 ± 5.73 | >50 |
|  | Rv2 | >50 | >50 |
|  | Rv3 | >50 | >50 |
|  | Rv4 | >50 | >50 |

| <1.0 | 1.01-10.0 | 10.01-50.0 | >50 | (µg/mL) |
| --- | --- | --- | --- | --- |

Note: Neutralization values are represented by a color scale on the right side of the table. Data were plotted as mean ± SEM (n = 3). HI, hemagglutination inhibition; MN, microneutralization.

**Table S4. Primers used in this study.**

| **Primer** | **sequences (5′-3′)** |
| --- | --- |
| Rv1-HA1bac-F | GAAGCGCGCGGAATTCAAAGGATGGTAAGCGCTATTGTTTTATATGTGCTTTTGGCGGCGGCGGCGCATTCTGCCTTTGCGGCGGACAAAATCTGCCTCGGACAT |
| Rv1-HA1bac-R | CTAGTGAGCTCGTCGACGTAGGTTAGTGATGGTGATGATGATGATGATGTCTTCCCTTTGGAATCTCAGGAAC |
| pFastBac-Dual-F | CCTACGTCGACGAGCTCACTAG |
| pFastBac-Dual-R | CCTTTGAATTCCGCGCGCTTC |
| CALL001 | GTCCTGGCTGCTCTTCTACAAGG |
| CALL002 | GGTACGTGCTGTTGAACTGTTCC |
| VHH-Forward | TTCCACCCAAGCAGTGGTATCAACGCAGAGTGGGAGTCTGGRGGAGG |
| VHH-Reverse | GTATCGATGCCCACCCTCTAGAGGCCGAGGCGGCCGACATGGAGACGGTGACCWGGGT |
| pGADT7-Rec-F | ATGTCGGCCGCCTCGGCCTCTAGAGGGTGGGCATCGATAC |
| pGADT7-Rec-R | CCACTCTGCGTTGATACCACTGCTTGGGTGGAA |
| GAL4AD-F | TACCACTACAATGGATG |
| 3AD-R | AGATGGTGCACGATGCACAG |
| bait-HA1-F | CTGCATATGGCCATGGAGGCCGAATTCGACAAAATCTGCCTCGGACAT |
| bait-HA1-R | ATGCGGCCGCTGCAGGTCGACGGATCTTCCCTTTGGAATCTCAGGAAC |
| pGBDT7 -F | GGATCCGTCGACCTGCAGCGGCCGCAT |
| pGBDT7 -R | GAATTCGGCCTCCATGGCCATATGCAG |
| 5α-Factor-F | TACTATTGCCAGCATTGCTGC |
| 3AOX1-R | GGCAAATGGCATTCTGACAT |
| Rv1/2/3/4-P1-up | CCTCCGAAGTTGGGGGGGAAGCAAAAGCAGG |
| Rv1/2/3/4-P2-down | TTGGGCCGCCGGGTTATTAGTAGAAACAAGG |
| F125A-P1-down | TTCTTATTCCACTGTATGTGGCTCCCATTG |
| F125A-P2-up | ACATACAGTGGAATAAGAACTAATGGAGCAACC |
| LHN-F | AGAATTCGAGCTCGGTACCC |
| LHN-R | GTGGCGTTTTTGGGGACAGGT |

In primers, K = G or T, W = A or T, R = A or G, Y = C or T, and M = A or C

**Table S5. Plasmid used in this study.**

| **Plasmid** | **Relevant characteristic(s)** | **Source** |
| --- | --- | --- |
| pDZ | reverse-genetics vector, Amp^R^ | Laboratory collection |
| pFastBac Dual | Expression vector, Gm^R^, Amp^R^ | Thermo |
| pPICZαA | Expression vector, Zeo^R^ | Invitrogen |
| pGADT7-Rec | Expression vector, Kan^R^ | Clontech |
| pGBKT7 | Expression vector, Amp^R^ | Clontech |
| pGBKT7-p53 | Positive control vector, Kan^R^ | Clontech |
| pGADT7-T | Positive control vector, Amp^R^ | Clontech |
| pGBKT7-Lam | Negative control vector, Kan^R^ | Clontech |

**Table S6. Bacterial and yeast strains used in this study.**

| **Strain** | **Relevant characteristic(s)** | **Source** |  |
| --- | --- | --- | --- |
| ***E. coli* Strains** | |  |  |
| DH5α | *deoR endA1 gyrA96 hsdR17 (rk-mk+) recA1 relA1 supE44 thi-1 Δ(lacZYA-argF) U169 Φ80lacZ ΔM15F - λ -* | Vazyme |  |
| DH10Bac | *F-, mcrA ∆(mrr-hsdRMS-mcrBC) ϕ80lacZ∆M15 ∆lacX74 recA1 endA1 araD139 ∆ (ara, leu)7697 galU galK λ- rpsL nupG /pMON14272 / pMON7124, Tet^R^ Kan^R^* | Coolaber |  |
| ***Pichia Pastoris* strains** | | |  |
| X33 | Wild-type strain | Laboratory collection |  |
| ***Saccharomyces cerevisiae* strains** | | | |
| Y187 | *MATα, ura3-52, his3-200, ade 2-101, trp 1-901, leu 2-3, 112, gal4Δ, met-, gal80Δ, URA3 :: GAL1UAS-GAL1TATA-lacZ,MEL1* | Clontech |  |
| Y2HGold | *MATa, trp1-901, leu2-3, 112, ura3-52, his3-200, gal4Δ, gal80Δ, LYS2 :: GAL1UAS-Gal1TATA-His3, GAL2UAS-Gal2TATA-Ade2 URA3::MEL1UAS-Mel1TATA AUR1-C MEL1* | Clontech |  |

**Figure S1. Comprehensive quality assessment of the yeast two-hybrid nanobody library and validation of bait functionality and recombinant protein expression.** A. Titer determination of the yeast two-hybrid (Y2H) nanobody library. A 10 µL aliquot of the library diluted 10⁵-fold was plated on SD/–Leu agar plates, yielding an average of 30.5 colonies per plate in duplicate experiments. The calculated library titer was approximately 3.05 × 10⁸ CFU/mL. SD/–Leu indicates synthetic dropout medium lacking leucine. B-C. Evaluation of insertion efficiency of the Y2H nanobody library. Twenty-five randomly selected colonies were analyzed by colony PCR, showing inserts of approximately 750 bp in all samples. 24 unique sequences were identified, including 23 in frame with the activation domain. M indicates the DL2000 DNA marker. D. Assessment of self-activation and toxicity of the bait construct in yeast. No detectable self-activation was observed, as indicated by white colonies on SD/–Trp, white colonies on SD/–Trp/X-alpha-Gal medium and lack of growth on SD/–Trp/X-alpha-Gal/AbA plates. No apparent toxicity was detected, as colony size was comparable to that of the empty vector control. SD/–Trp, synthetic defined medium lacking tryptophan; SD/–Trp/X-α-Gal, SD/–Trp supplemented with X-α-Gal; SD/–Trp/X-α-Gal/AbA, SD/–Trp supplemented with X-α-Gal and aureobasidin A (AbA). E. Identification of positive interactions in the Y2H system. Representative images of mated yeast diploids containing both bait and prey plasmids were plated on QDO/X/A (quadruple dropout medium supplemented with X-α-Gal and Aureobasidin A). Blue colonies indicate activation of reporter genes and represent positive yeast clones with specific protein-protein interactions. F-G. SDS-PAGE analysis of purified nanobodies and HA1 proteins. The nanobodies were produced in *P. pastoris* yeast strain X33 and HA1 proteins produced using a baculovirus–insect cell system. The nanobodies and HA1 proteins exhibited expected molecular weights of ~15 kDa and ~40 kDa, respectively, with purities exceeding 95%
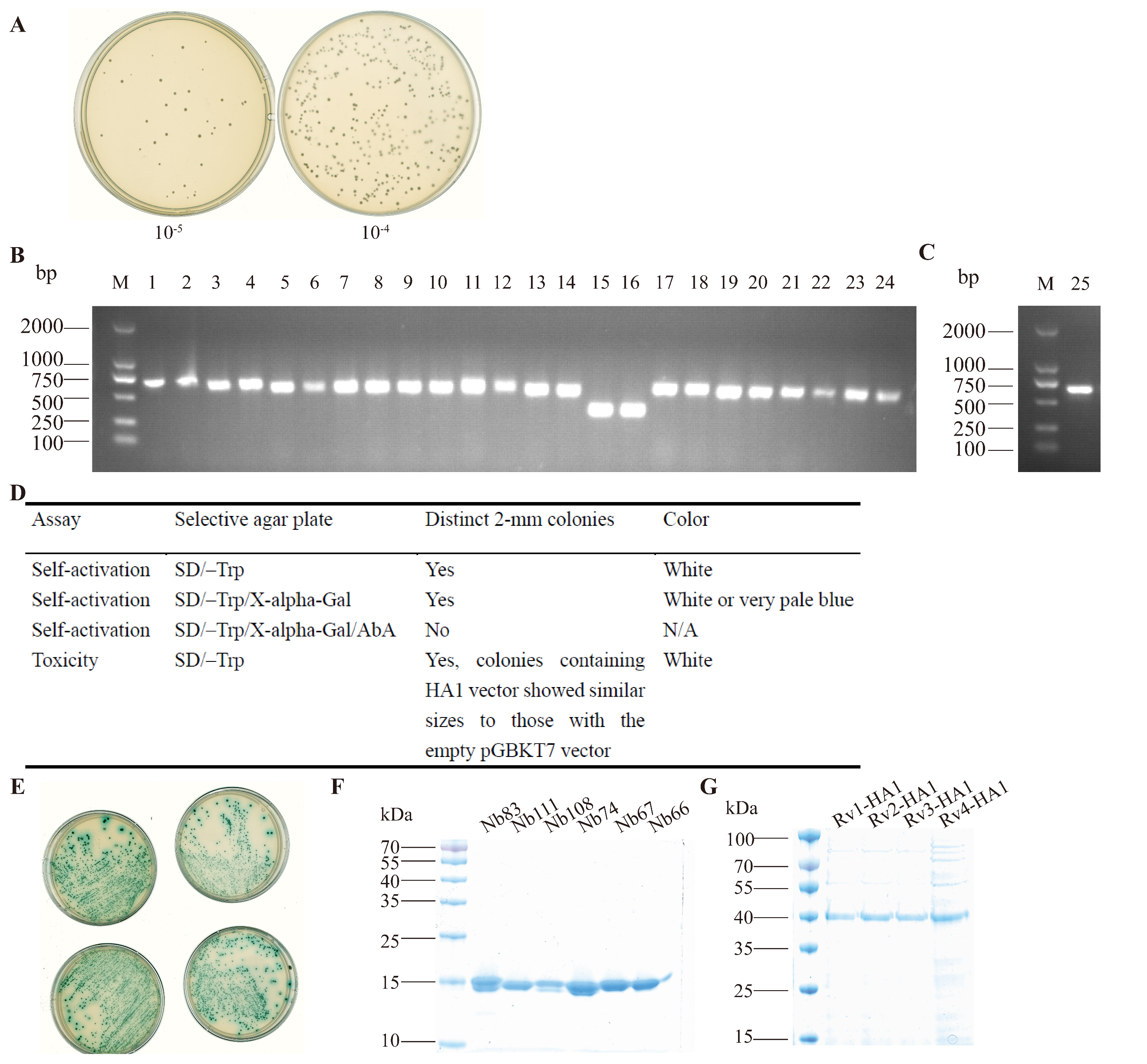
and ~90%, as shown on 15% and 12% gels. Molecular weight markers (kDa) are indicated.

**Figure S2.** **Raw SPR sensorgrams of Nb74 binding to Rv1-Rv4 HA1 proteins.** A-D. The response is plotted as response units (RU) versus time (s). Nb74 was injected at the indicated concentrations shown in the upper right corner of each panel. These traces represent the unprocessed experimental data without curve fitting; the corresponding fitted sensorgrams and kinetic analyses are presented in the main text.


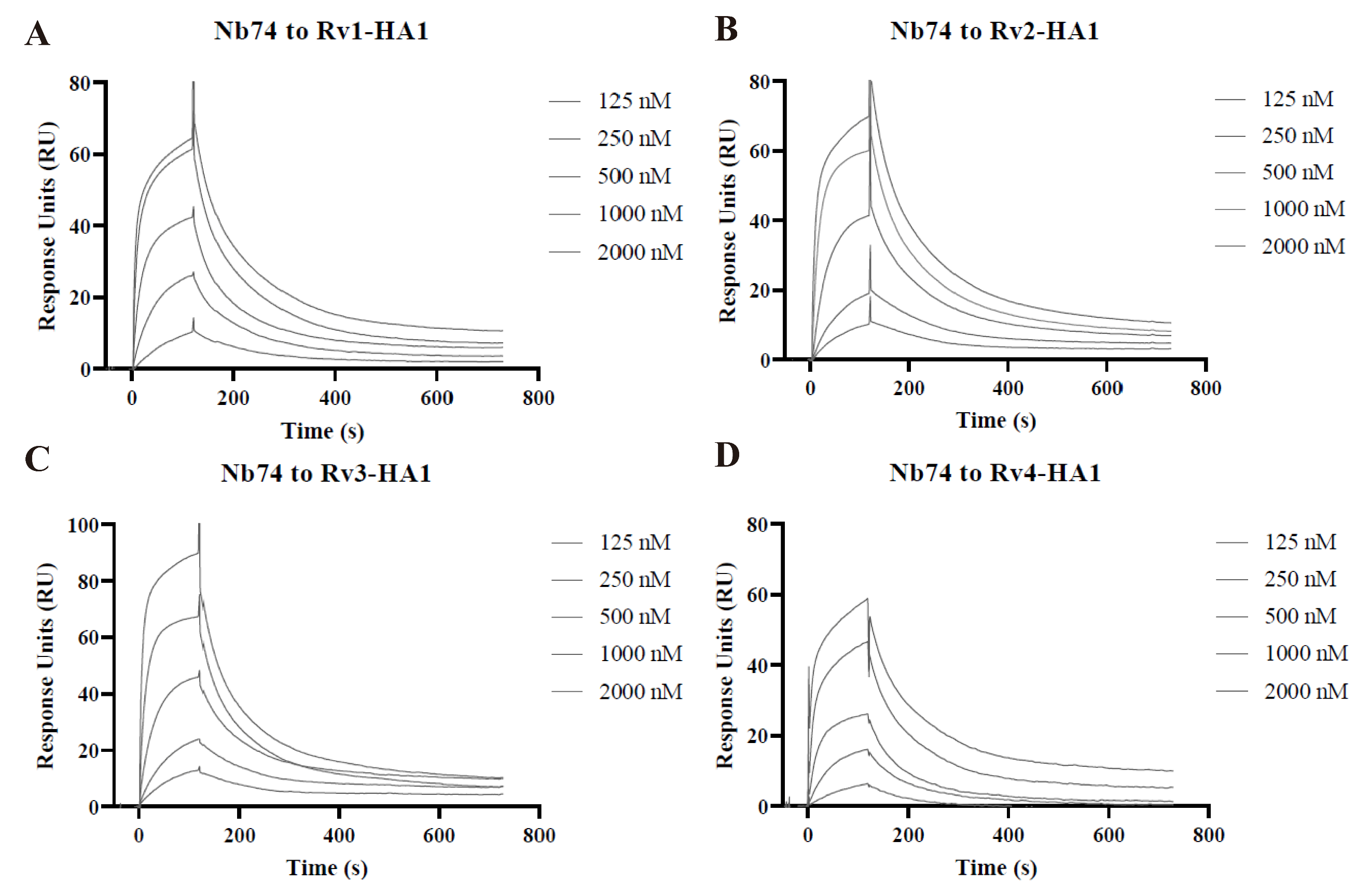


**Figure S3. Structural interpretation of Nb74 epitope suggests a potential quaternary binding mode.** A. Peptide coverage and quality assessment for HDX-MS analysis of HA1. A peptide coverage map of HA1 showing the distribution of identified peptides along the protein sequence. Red bars represent individual peptides retained after filtering. From an initial set of 4,222 peptides, 178 high-confidence peptides were selected based on the following criteria: minimum intensity of 5,000, at least 0.3 fragment ions per amino acid, minimum identification score of 7, and a maximum precursor mass error of 10 ppm. These filtering parameters resulted in an estimated sequence coverage of 97.9% and an average redundancy of 8.49 for covered residues. The bottom panel summarizes the final dataset, showing that 99 peptides provide 98.2% sequence coverage with an average redundancy of 4.34. B. Spatial proximity of the 219-277 peptide to escape-associated residues across adjacent HA protomers. HDX-protected peptides are mapped onto the HA trimer, with representative deuterium uptake kinetics shown (mean ± SD). The 219-277 peptide and escape-associated residues are highlighted. Although separated within a single protomer, these regions are positioned in close spatial proximity across neighboring HA protomers in the structural model. C. Putative epitope spanning adjacent HA protomers. Surface representation of the HA trimer in complex with Nb74, highlighting the predicted binding site at the interface between adjacent protomers. Nb74 spans neighboring subunits and may interfere with receptor access to the receptor-binding site (RBS) on an adjacent HA protomer.

**
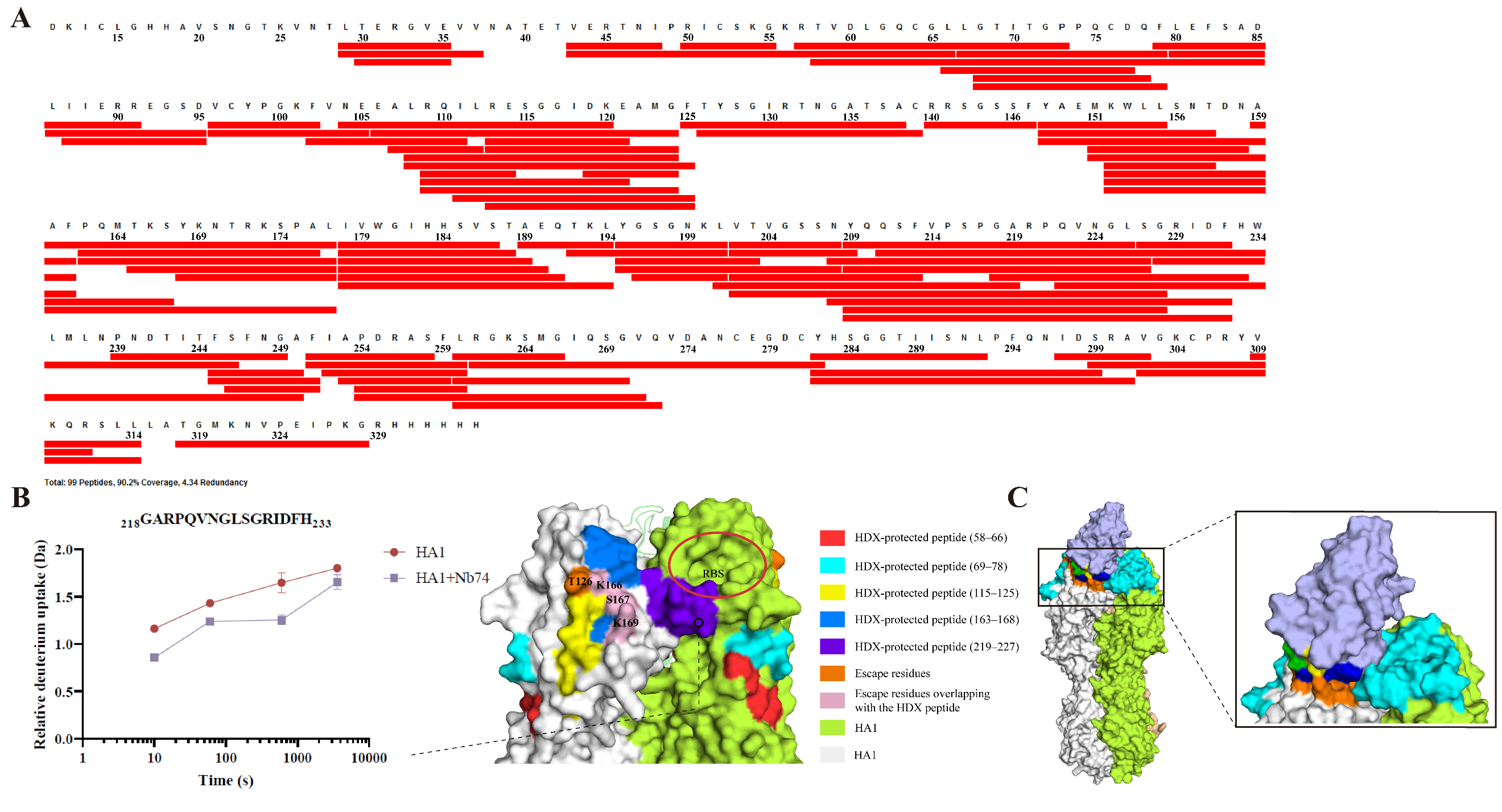
**
